# The roles of barriers, refugia, and chromosomal clines underlying diversification in Atlantic Forest social wasps

**DOI:** 10.1038/s41598-017-07776-7

**Published:** 2017-08-09

**Authors:** Rodolpho S. T. Menezes, Seán G. Brady, Antônio F. Carvalho, Marco A. Del Lama, Marco A. Costa

**Affiliations:** 1 Departamento de Biologia, Faculdade de Filosofia, Ciências e Letras - Universidade de São Paulo (FFCLRP/USP), Av. Bandeirantes, 3900, 14040-901 Ribeirão Preto, SP Brazil; 2 Departamento de Ciências Biológicas, Universidade Estadual de Santa Cruz, Rodovia Jorge Amado, Km 16, 45662-900 Ilhéus, BA Brazil; 3 Department of Entomology, National Museum of Natural History, Smithsonian Institution, Washington, DC 20560-0188 USA; 4 Departamento de Genética e Evolução, Universidade Federal de São Carlos, Rodovia Washington Luís, Km 235, 13565-905 São Carlos, SP Brazil

## Abstract

Phylogeographic studies have sought to explain the genetic imprints of historical climatic changes and geographic barriers within the Brazilian Atlantic Forest (AF) biota, and consequently two processes of diversification (refugia and barriers) have been proposed. Additionally, there is evidence that eustatic changes influenced the biogeographic history of the AF. Here we evaluate these contrasting diversification processes using two AF social wasp species – the mid-montane *Synoeca cyanea* and the lowland *Synoeca* aff. *septentrionalis*. We analyzed several sources of data including multilocus DNA sequence, climatic niche models and chromosomal features. We find support for idiosyncratic phylogeographic patterns between these wasps, involving different levels of population structure and genetic diversity, contrary suitable climatic conditions during the last glaciation, and contrasting historical movements along the AF. Our data indicate that neotectonics and refugia played distinct roles in shaping the genetic structure of these wasps. However, we argue that eustatic changes influenced the demographic expansion but not population structure in AF biota. Notably, these wasps exhibited chromosomal clines, involving chromosome number and decreasing of GC content, latitudinally oriented along the AF. Together, these results reinforce the need to consider individual organismal histories and indicate that barriers and refugia are significant factors in understanding AF evolution.

## Introduction

Intense climatic swings during the late Quaternary had notable effects on the population dynamics, range and genetic diversity of many organisms^1^. Nevertheless, the magnitude of these biological effects during glaciations is generally dependent on the species’ environmental requirements, vagility^2, 3^, latitude and topography^1, 4^. Multiple investigations have shown the intrinsic role of glacial-interglacial cycles for northern hemisphere taxa^5, 6^, revealing a process of range contraction during glaciation^7^. Also, studies on tropical organisms such as Australian and African forest-dwelling species have revealed range contraction during the Last Glacial Maximum (LGM), around 21 thousand years ago (21 kya)^8–11^. However, range and population expansions even during the LGM have been proposed for some cold/arid-tolerant biota^4, 12, 13^.

The high biotic diversity within Neotropical forests presents a conundrum that has challenged evolutionary and biogeographic biologists to explain the cause of these diversity patterns in such environments. Hence, particular models considering historical mechanisms capable of promoting diversification have been invoked to explain the diversity pattern in the eastern South America, particularly the Brazilian Atlantic Forest (hereafter AF). The model of climatically stable habitat through time in the AF, proposed by Carnaval & Moritz^14^ and Carnaval *et al*.^15^ (hereafter CM model), suggests that climatically stable areas (e.g., refugia), even during the LGM, were restricted to its northern areas (above São Francisco river; Pernambuco refuge) and central areas (located between the São Francisco and Doce rivers; Bahia refuge), whereas the southern portion of the AF would have been climatically unstable. Moreover, the north and south AF represent two distinct climatic regions^16^, reinforcing the idea that these two geographic areas provided different paths to the diversification history of AF species. Notably, several phylogeographic studies are consistent with the CM model in showing higher genetic diversity, strong population structure and an absence of demographic expansion signals within populations in the north and central portion of the AF, whereas populations in the southern portion show lower genetic diversity, signals of demographic expansion (due to recent re-colonization events from north populations) and weaker population structuring^14, 15, 17–21^.

However, other studied species reveal genetic responses contrary to that expected by the CM model, suggesting instead a meaningful role for geographic barriers^22–25^, including the newly proposed influence of sea-level changes on the biogeographic history of the AF^26^. In contrast to refugia scenarios, genetic predictions regarding barrier hypotheses include older divergence times (due to vicariant events occurring deeper in the past), interruptions of gene flow that coincide with geographic barriers, and populations showing smooth demographic oscillations^27^. Another alternative hypothesis to the phylogeographic patterns observed in AF species, termed the “Atlantis Forest hypothesis”, suggests that populations actually expanded, even during the LGM, in response to the expansion of the AF onto the Brazilian emerged continental shelf^26^.

**Figure 1:**
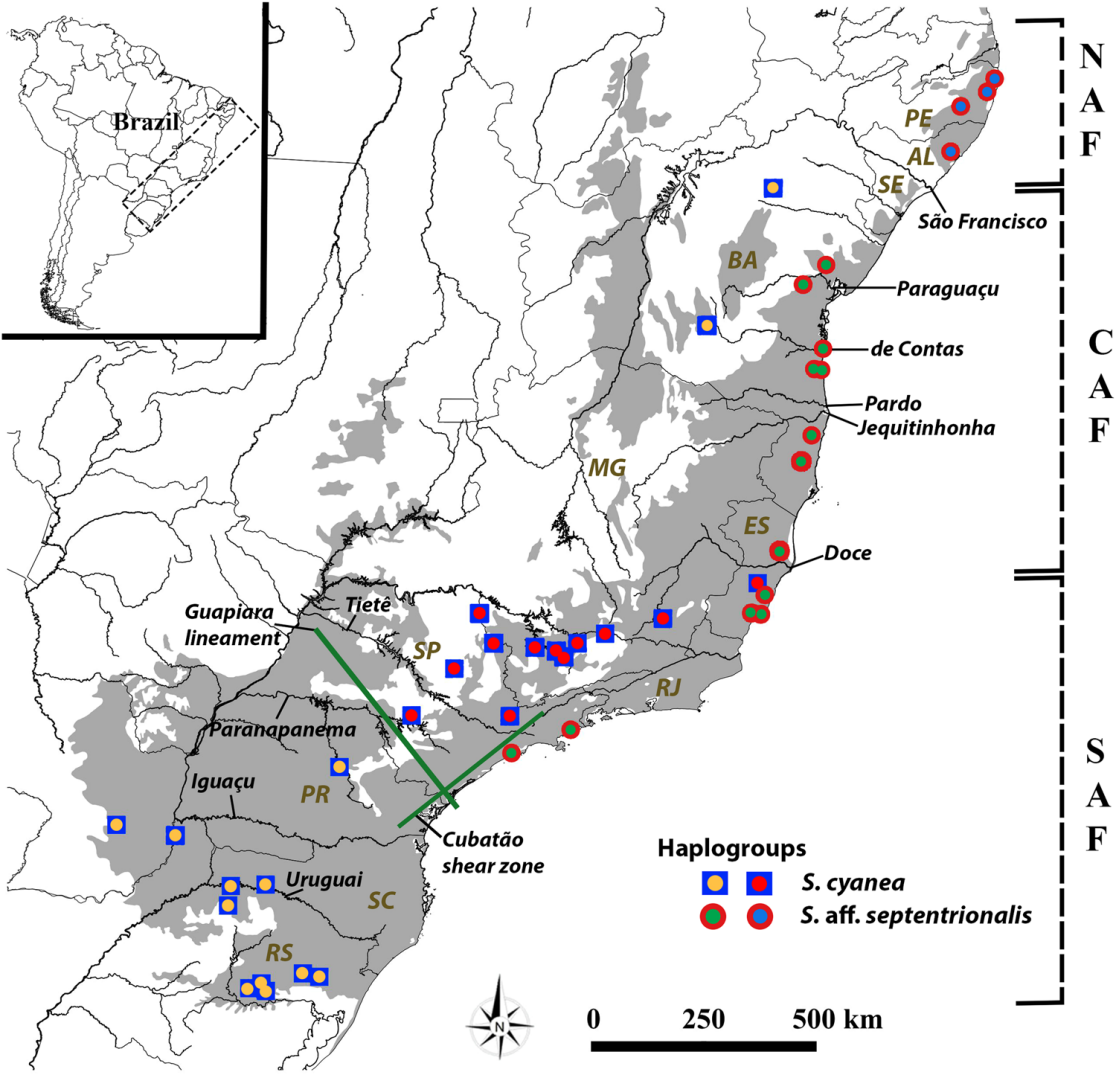
Map showing collection sites in the present study (see also Supplementary Table S1). Atlantic Forest (AF) is indicated in light grey, state borders in grey and main rivers (São Francisco, Paraguaçu, de Contas, Pardo, Jequitinhonha, Doce, Tietê, Paranapanema, Iguaçu and Uruguai) in black. Four haplogroups are indicated (see text for details). Schematic subdivision of the AF along its geographical range: North Atlantic Forest (NAF), Central Atlantic Forest (CAF) and Southern Atlantic Forest (SAF). Brazilian states: Rio Grande do Sul (RS), Santa Catarina (SC), Paraná (PR), São Paulo (SP), Rio de Janeiro (RJ), Espírito Santo (ES), Minas Gerais (MG), Bahia (BA), Sergipe (SE), Alagoas (AL) and Pernambuco (PE). Green lines represent areas of putative geographic barriers influencing *S. cyanea* distribution. The map was generated using the software Quantum-GIS v2.8 (Open Source Geospatial Foundation Project, Beaverton, OR, USA) (http://www.qgis.org/en/site/forusers/download.html).

We evaluated these models using two species of Neotropical swarm-founding wasps within the genus *Synoeca* (Vespidae, Polistinae, Epiponini). These are aggressive, medium-sized social wasps with black or metallic colours that build nests directly attached to tree branches^28^. In Brazil, these wasps are popularly known as “*marimbondo* (or *caba*) *tatu*”, meaning “armadillo” wasp, because the envelope of their nests resembles the back of an armadillo (Dasypodidae). The mid-montane *Synoeca cyanea* (Fabricius, 1775) inhabits the southern and central portions of AF; it is found regularly in semi-deciduous and gallery forests, showing a strong trend for mid-elevation habitats in lower latitudes, reaching up to 1100 meters above sea level (a.s.l.)^28, 29^ (Fig. 1). Meanwhile, the lowland *S*. aff. *septentrionalis* (≤450 m a.s.l) is restricted to the AF coastline, occupying dense ombrophilous forest and occasionally restinga habitat (see Menezes *et al*.^29, 30^) (Fig. 1). The two wasps possess similar behaviors (e.g., swarming, nesting and reproduction), generation times, vagility (i.e., due to similar size these wasps have similar capacity for relatively long-distance dispersal), and sensitivities to climate change^31^, but remain ecologically distinct due to their putative thermal adaptations. Hence, these social insects are promising candidates to test the diversification models (e.g., refugial, barriers and Atlantis hypotheses) proposed for AF biota.

The combination of phylogeographic analysis and ecological niche modeling (ENM) has proven to be a powerful approach providing remarkable insights regarding microevolutionary processes^13, 15, 26, 32^. On the other hand, only a few AF phylogeographic studies have explored chromosome data, despite karyotypic differences having the potential to act as a genetic barrier^33, 34^, and consequently might reveal strong evidence of population structuring (e.g., Amaro *et al*.^23^). Here, we used independent data including multilocus DNA sequence, climatic niche models, and chromosomal features to investigate and compare the phylogeographic patterns of these two parapatric social wasps. Additionally, we explored whether these armadillo wasps experienced the scenarios proposed by the contrasting diversification models suggested for AF biota, and also evaluated the potential role of the Brazilian emerged continental shelf during the LGM on their demographic histories. Specifically, if multiple processes (e.g., refugia and barriers) drove the diversification and historical demography in AF biota, we expect that mid-montane (*S. cyanea*) and lowland (*S*. aff. *septentrionalis*) species would have experienced different patterns of historical population dynamics, which would argue against a single dominant explanation for diversification within this region. Thus, we first compared levels of population structure, historical demographic change, and suitable climatic conditions during the LGM to assess the roles of barriers and refugia in shaping the genetic structure of AF social wasps. Second, assuming that the north and south AF could act as distinct climatic regions, we inferred the putative geographic origin and historical movements along the AF for these species. Finally, we collected chromosomal data in search for additional evidence of phylogeographic structure and the directionality of their population dispersal.

## Materials and Methods

### Sampled specimens

Samples from each species were collected between 2010 and 2013, supplemented by additional material from museum specimens. Sampling was conducted from multiple localities (*S*. *cyanea*, n = 37 from 25 localities; *S*. aff. *septentrionalis*, n = 24 from 17 localities) with the aim of covering their respective ranges (see Fig. 1, Supplementary Table S1). All field-collected specimens were preserved in ethanol at −20 °C prior to the molecular analyses. Vouchers are deposited in the entomological collections at the Coleção Entomológica ‘J.M.F. Camargo’ (RPSP) (FFCLRP-USP, São Paulo, Brazil) and the National Museum of Natural History (USNM; Smithsonian Institution; Washington, DC, USA). The collection of samples in Brazil was carried out in agreement with the appropriate collection permits from the Brazilian Institute for Biodiversity Conservation (ICMBio) (SISBio number 22180). Species-level taxonomy was based on the identification keys provided by Richards^28^ and Andena *et al*.^35^, as well as including the recommendations regarding external morphological structures proposed by Menezes *et al*.^29^.

### DNA extraction, amplification, Sanger sequencing and sequence edition

We removed thorax or hind leg tissues from each specimen sampled (one individual sample per nest) and extracted total genomic DNA using the DNeasy Blood & Tissue Kit (Quiagen Inc., Valencia, California, U.S.A.). We amplified multiple genetic loci as follows: mitochondrial (mtDNA) fragments of 16 S ribosomal DNA (16 S), 12 S ribosomal DNA (12 S), cytochrome oxidase subunit I (COI), cytochrome oxidase subunit II (COII), cytochrome b (CytB), and nuclear (nDNA) fragments of carbamoylphosphate synthetase (CAD) and elongation factor 1-alpha F2 copy (EF1α) (Table 1). The genomic fragments were amplified by standard PCR employing specific primers and amplification conditions as outlined in Supplementary Table S2. Nuclear protein-coding loci (CAD and EF1α) were selected due to the known presence of introns within Hymenoptera^36–38^. Sequencing reactions for each PCR product were performed with same PCR primers and BigDye® Terminator ver. 3.1 Cycle Sequencing on ABI 3730 Sequencers© (Life Technologies, Frederick, MA). Each bidirectional sequence was assembled into contigs and edited using Sequencher v.5.2.4 (Gene Code Corp., Ann Arbor, MI, USA). Multiple sequence alignments were generated using Muscle 3.7^39^ implemented in MEGA version 6.06^40^. We checked for recombination in each protein-coding gene using the pairwise homoplasy index (PHI) test implemented in SplitsTree v4.14.2^41^.

**Table 1:** Genetic diversity and neutrality tests for the two armadillo wasps. Sample size (n), nucleotide diversity in percent (%π), number of polymorphic/segregating sites (*S*), number of haplotypes (H), haplotype diversity in percent (%Hd), Tajima’s *D* (*D*), Fu’s *Fs* (*Fs*), and Ramos-Onsins and Rozas (*R*_2_).

*Synoeca cyanea*		Summary statistics						Neutrality tests		
Locus	Haplogroup	bp	n	*S*	H	%Hd	%π	*D*	*F*s	*R*_2_
**16S**	All	508	35	7	8	71	0.28	—	—	—
	NGL		20	5	4	28	0.13	−1.58577*	−0.8819	0.1026
	SGL/non-Bahia		13	4	4	65	0.22	−0.42367	−0.2530	0.1681
**CytB**	All	433	35	15	15	86	0.44	—	—	—
	NGL		19	10	7	70	0.35	−1.83866**	−2.1432	0.1211
	SGL/non-Bahia		13	6	7	73	0.24	−1.6849*	−4.5638**	0.0844**
**COI**	All	472	25	30	16	91	1.39	—	—	—
	NGL		17	9	9	82	0.50	−0.3788	−3.1892*	0.1245
	SGL/non-Bahia		5	22	5	100	2.67	1.43249	−0.0393	0.2616
**COII**	All	629	22	6	7	73	0.16	—	—	—
	NGL		16	4	5	70	0.15	−0.5765	−1.4689	0.1234
	SGL/non-Bahia		4	1	2	50	0.08	−0.6124	0.1719	0.4330
**12S**	All	354	21	0	1	0	0.00	—	—	—
	NGL		15	0	1	0	0.00	0.0000	0.0000	0.0000
	SGL/non-Bahia		4	0	1	0	0.00	0.0000	0.0000	0.0000
**CAD**	All	361	22	1	2	24	0.06	—	—	—
	NGL		17	1	2	30	0.08	0.08512	0.50675	0.1544
	SGL/non-Bahia		3	0	1	0	0.00	0.0000	0.0000	0.0000
**EF1α**	All	873	21	0	1	0	0.00	—	—	—
	NGL		17	0	1	0	0.00	0.0000	0.0000	0.0000
	SGL/non-Bahia		2	0	1	0	0.00	0.0000	0.0000	0.0000
***Synoeca*** **aff**. ***septentrionalis***		**Summary statistics**						**Neutrality tests**		
**Locus**	Haplogroup	bp	n	*S*	H	%Hd	%π	*D*	*F*s	*R*_2_
**16S**	All	508–509	24	17	8	84	0.75	—	—	—
	NAF		5	15	3	70	1.45	0.203087	3.12501	0.2092
	CSAF		19	4	5	76	0.28	−0.00797	−0.28024	0.1509
**CytB**	All	433	23	25	12	79	0.94	—	—	—
	NAF		4	16	4	100	1.84	−0.8490	0.0687	0.2931
	CSAF		19	9	8	67	0.26	−1.9675**	−4.6545**	0.0765**
**COI**	All	472	23	24	13	89	0.94	—	—	—
	NAF		5	15	3	70	1.80	0.7827	3.4319	0.2400
	CSAF		19	12	11	86	0.40	−1.9384**	−6.6560**	0.0771**
**COII**	All	629	21	23	11	89	0.86	—	—	—
	NAF		4	14	3	83	1.11	−0.8453	2.0379	0.3928
	CSAF		17	8	8	84	0.32	−0.5256	−2.6359*	0.1182
**12S**	All	354	16	8	3	24	0.28	—	—	—
	NAF		2	8	2	100	2.26	—	—	—
	CSAF		14	0	1	0	0.00	0.0000	0.0000	0.0000
**CAD**	All	361	21	6	7	78	0.31	—	—	—
	NAF		4	2	2	50	0.27	−0.7099	1.0986	0.4330
	CSAF		17	6	6	69	0.27	−1.5197*	−2.6502*	0.1002*
**EF1α**	All	873	20	2	3	28	0.05	—	—	—
	NAF		3	1	2	66	0.07	—	—	—
	CSAF		17	0	1	0	0.00	0.0000	0.0000	0.0000

p < 0.05 (*); p < 0.01 (**).

### Investigation of population structure, phylogenetic relationships and genetic diversity

Relationships among haplotypes and their spatial distribution were assessed using median-joining networks^42^ implemented in the program Network 4.613 (www.fluxus-engineering.com). We defined haplogroups within the context of putative geographic barriers proposed for the AF within the range of both armadillo wasp species (see results section). Measures of genetic differentiation (based on average number of nucleotide differences per site; *Dxy*) among haplogroups were estimated for each locus using DnaSP v5.10.1^43^. We also investigated the degree of genetic structure among haplogroups, among localities within haplogroups, and within localities using analysis of molecular variance (AMOVA; Excoffier *et al*.^44^) in Arlequin 3.5.1.2^45^ with 10,000 iterations.

Prior to phylogenetic inference, alignments for each locus were concatenated and codon positions delimitated using Mesquite v3.02^46^. The most appropriate partition scheme and the best fitting model for each partition were selected using PartitionFinder v1.1.1^47^ under the Bayesian information criterion (BIC). Bayesian Inference (BI) was conducted using MrBayes v3.2.3^48^ with two independent runs of 50 million generations each with four chains (temp = 0.1) and sampled every 10,000 generations. Tracer v1.6^49^ was used to assess convergence in the Markov chains by considering the effective sample size (ESS > 200). We removed as burn-in the first 20% of sampled generations and produced a maximum credibility tree. Trees were visualized and edited using FigTree v1.4.2.

To compare the amount and patterns of genetic diversity between the two armadillo wasp species, we calculated the nucleotide diversity (π), number of haplotypes (H), haplotype diversity (Hd) and number of polymorphic/segregating sites (*S*) for each locus using DNAsp v5.10.1^43^.

### Tests for historical demographic change

Demographic history was inferred based on the neutrality tests and assumptions of constant population size using the Tajima’s *D* ^50^, Fu’s *Fs* ^51^ and *R*_2_ statistics^52^ in DNAsp v5.10.1^43^ for each locus, with 10,000 coalescent simulations to calculate significance values.

Additionally, we reconstructed the historical demography over time for each haplogroup using the Extended Bayesian Skyline Plot method (EBSP; Heled & Drummond^53^) implemented in BEAST v1.8.2^54^. This coalescent method increases the precision to infer demographic dynamics, even with a small number of individuals, by incorporating multiple unlinked loci rather than the use of a single locus^53, 55^. We selected a strict clock for each EBSP run based on haplogroup-level structure within each species. We also specified a substitution rate for mtDNA sequences at 1.2–1.5% per million years calibrated to other insects^56^, and estimated substitution rates for the nDNA data set. Analyses were run with a random tree and linear model. We performed two replicate runs with 100 million of generations, with trees sampled every 10,000 generations. Tracer v1.6^49^ was used to verify convergence through ESS values (>200). Replicate runs were combined after a burn-in of 10% using LogCombiner v1.8.2.

### Ecological niche modeling (ENM) as test for range expansion, contraction or shift

To identify present day climate-suitable habitat for both species of armadillo wasps, we combined georeferenced occurrence data points [*S*. *cyanea*: 68 points; *S*. aff. *septentrionalis*: 19 points (see Supplementary Table S3)] and 19 bioclimatic variable layers (2.5-min resolution)^57^ available through the WorldClim database (http://www.worldclim.org) using the maximum entropy algorithm in MAXENT v3.3.3k^58, 59^. Bioclimatic variable layers were limited to eastern South America. Occurrence points were gathered through specific literature, surveying, specimens donated, loaned and/or examined from entomological collections from museums or universities listed in the Acknowledgements section (see also Supplementary Table S3). As incorrect (or incomplete) taxonomy can affect the accuracy of niche modeling^60^, we avoided occurrence points from online databases.

Initially, we verified and excluded correlated bioclimatic layers based on Principal Component Analysis (PCA) using PAST v3.02^61^. For this specific step, we extracted values of each occurrence point associated to each 19 bioclimatic layers using Quantum-GIS v2.8 (Open Source Geospatial Foundation Project, Beaverton, OR, USA) and we checked auto-correlated variables by PCA. Simultaneously, we verified the percentage contributed to each bioclimatic layer and we excluded the variables with non-contributions. The models were run with the following parameters: quadratic, product, threshold and hinge, 500 iterations, regularization multiplier equal 1, thirty percent of random test and 10 replicates subsampled. Finally, present-day ENMs were projected into bioclimatic variables predicted for two different past scenarios, Last Interglacial (LIG; 120–140 kya) and LGM (21 kya) from MIROC (Model of Interdisciplinary Research on Climate) and CCSM3 (Community Climate System Model) models.

### Tracking historical movements

To infer the historical movements of both species, we applied a Bayesian phylogeographic approach under a relaxed random walk (RRW model) diffusion model across continuous space in BEAST v1.8.2^54^ using the mtDNA-only data set. This model considers heterogeneous rates in a diffusion process across the branches of the phylogeny^62^ and allows for probabilistic inference of historical movements. We used the Jitter option (with 0.5) to add random noise to samples with similar coordinates. We applied a GMRF skyride model as demographic prior for RRW analyses and mtDNA substitution rate estimated as applied in EBSP analyses (see above).

We performed four replicate runs of 200 million generations each and sampled at every 10,000 steps. Tracer v1.6^49^ was used to verify convergence through ESS values (>200). Replicate runs were combined after a burn-in of 10% using LogCombiner v1.8.2, and the maximum clade credibility tree was accessed using TreeAnnotator v1.8.2. Posteriorly, we generated a visual representation of the historical movements experienced by these armadillo wasps in Google Earth v7.1.5 (Google Inc.) using the Continuous Tree module in SPREAD v1.0.6^63^.

### Chromosomal features

Mitotic metaphases were obtained from neural ganglia cells during the third instar of larvae according to Imai *et al*.^64^. Only sampling events that preserved live larvae were available for cytogenetic analysis (see Table 2 for sampling information). Chromosomes were stained with Giemsa® to visualize the chromosome morphology. In order to locate GC/AT-rich chromosomal segments, we applied fluorochrome staining (chromomycin A_3_ [CMA_3_]/4,6-diamidino-2-phenylindole [DAPI]) following Schweizer^65^ with modifications proposed by Guerra & Souza^66^. Due to few slides available for some populations, some slides were first stained with fluorochrome and subsequently stained with Giemsa®.

**Table 2:** Summary information about chromosomal features investigated from *S*. *cyanea* and *S*. aff. *septentrionalis*.

Species	Locality	H	N	2n (n)	k	Chromosomes^#^
*S. cyanea*	Senhor do Bonfim, BA (colony 1)	Bahia	12♀	42	14 M + 4SM + 2 A	1p^t^/1p^+^q^sb^, 2^b^p^t^, 3p^t^q^sb^, 4p^t^, 5^b^, 6^b^, 7p^t^q^sb^, 8^b^, 9 q^t^, 10^b^, 11^b^, 12^b^, 13^b^, 14^b^, 15p^t^, 16^b^, 17^b^, 18^b^, 19^b^, 20^b^, 21 q^t^
	Senhor do Bonfim, BA (colony 2)	Bahia	04♀	42	14 M + 4SM + 2 A	1p^t^/1p^+^q^sb^, 2^b^p^t^, 3p^t^q^sb^, 4p^t^, 5^b^, 6^b^, 7p^t^q^sb^, 8^b^, 9 q^t^, 10^b^, 11^b^, 12^b^, 13^b^, 14^b^, 15p^t^, 16^b^, 17^b^, 18^b^, 19^b^, 20^b^, 21 q^t^
	Santa Teresa, ES	NGL	04♀	40	11 M + 8SM	1p^t^q^sb,t^/1^b^, 2p^t^q^sb^, 3p^t^q^sb^/3^pc^p^t^, 4p^t^, 5p^t^q^sb,t^/5^+^, 6p^t^, 7p^t^, 8^b^, 9p^t^, 10p^t^, 11^b^, 12^b^, 13^b^, 14^b^, 15^b^, 16q^t^, 17^b^, 18^b^, 19^b^, 20q^t^
	Carlos Barbosa, RS	SGL	03♀	38	10 M + 9SM	1^pc^p^t^, 2^pc^p^+^, 3^pc^p^+^, 4^pc^p^t^, 5^b^, 6^pc^p^+^/6^pc^q^t^, 7^pc^q^+^, 8^b^, 9^b^, 10^b^, 11^b^, 12^b^, 13^b^, 14^b^, 15q^t^, 16^b^, 17q^t^, 18^+^, 19^b^
*S*. aff. *septentrionalis*	Santa Teresinha, BA	CSAF	08♀/03♂	41 (21)	10 M + 10SM + 1 A	1q^t^, 2^b^, 3^b^, 4^b^, 5^b^, 6^b^, 7^b^, 8^b^, 9^b^, 10^b^, 11^b^, 12^b^, 13^b^, 14^b^, 15^b^, 16^b^, 17^b^, 18q^t^, 19q^t^, 20q^t^, 21^b^/21q^t^
	Itabuna, BA	CSAF	09♀	41	10 M + 10SM + 1 A	1q^t^, 2^b^, 3^b^, 4^b^, 5^b^, 6^b^, 7^b^, 8^b^, 9^b^, 10^b^, 11^b^, 12^b^, 13^b^, 14^b^, 15^b^, 16^b^, 17^b^, 18q^t^, 19q^t^, 20q^t^, 21^b^
	Itacaré, BA	CSAF	16♀	41	10 M + 10SM + 1 A	1q^t^, 2^b^, 3^b^, 4^b^, 5^b^, 6^b^, 7^b^, 8^b^, 9^b^, 10^b^, 11^b^, 12^b^, 13^b^, 14^b^, 15^b^, 16^b^, 17^b^, 18q^t^, 19q^t^, 20q^t^, 21^b^
	Ilhéus, BA	CSAF	07♀	41	10 M + 10SM + 1 A	1q^t^, 2^b^, 3^b^, 4^b^, 5^b^, 6^b^, 7^b^, 8^b^, 9^b^, 10^b^, 11^b^, 12^b^, 13^b^, 14^b^, 15^b^, 16^b^, 17^b^, 18q^t^, 19q^t^, 20q^t^, 21^b^
	Vila Regência, ES	CSAF	07♀	38	8 M + 9SM + 2 A	1^b^, 2^b^, 3^b^, 4p^t^, 5^b^, 6^b^, 7^b^, 8^b^, 9^b^, 10^b^, 11^b^, 12^b^, 13^b^, 14^b^, 15^b^, 16^b^, 17^b^, 18^b^, 19^b^
	Moreno, PE	NAF	11♀	40	10 M + 9SM + 1 A	1^+^, 2^+^, 3^+^, 4^b^, 5^+^, 6^b^, 7^b^, 8^b^, 9^b^, 10^b^, 11^b^, 12p^t^, 13^b^, 14^b^, 15^b^, 16p^t^, 17^b^, 18^b^, 19q^t^, 20q^t^

H: haplogroups and Bahia haplotype (see text for details); N: number of specimens with chromosomes analyzed; k: karyotypic formulae; #: chromosome and specific position that CMA_3_^+^ sites are located; p: short arm; q: long arm; b: CMA_3_^+^ site in both arms; sb: subcentromeric; pc: pericentromeric; t: terminal position; + : entire arm.

Chromosomal observations and image capture were conducted using both epifluorescence photomicroscope Olympus BX51 equipped with a DP-72 digital camera and DMRA2 Leica (Leica Microsystems Imaging Solutions Ltd, Cambridge, UK). We observed a minimum of five metaphases per slide to assure unambiguous chromosome number counts. The best quality metaphase plate was used for karyogram preparation using Adobe Photoshop CS6 v13.0. Finally, the chromosomes were arranged in decreasing order of size and classified according to Levan *et al*.^67^.

## Results

### DNA sequence data

Our sequence data set contained a total of 3621–3622 base pairs (bp) composed of mtDNA protein-coding COI (472 bp − 13.04% of data set), tRNA-Leu + COII (47 + 582 bp − 17.37%), CytB (433 bp − 11.96%), mtDNA non-protein-coding 16 S (508–509 bp − 14.03%), 12 S (354 bp − 9.77%), and nDNA introns from CAD (361 bp − 9.97%) and EF1α (873 bp − 24.11%). No premature stop codons were found within protein-coding sequences. Half of the *S*. aff. *septentrionalis* sequences had a single base pair indel approximately in the middle of the 16 S sequences. The PHI test yielded no statistically significant evidence for recombination in each protein-coding gene. All sequences are deposited in GenBank and their respective accession numbers are presented in Supplementary Table S1.

### Phylogeographical structure, Bayesian phylogenetics and genetic diversity

**Figure 2:**
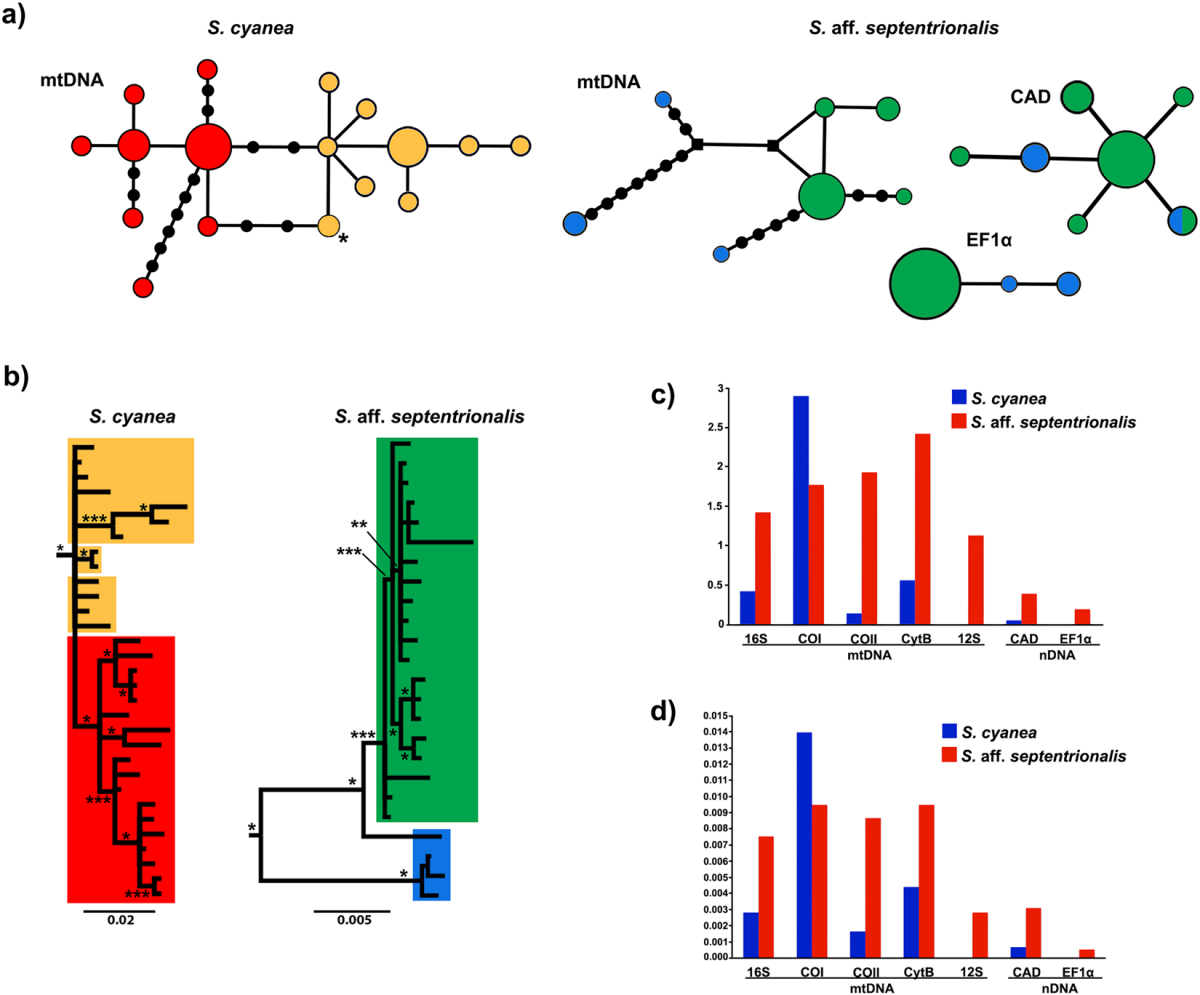
Phylogeographic structure and nucleotide diversity. (**a**) Unrooted network of mtDNA (based on 16 S + Cytb for *S. cyanea*; and based on 16 S + Cytb + COI + COII for *S*. aff. *septentrionalis*) and nDNA haplotypes. For *S*. *cyanea*, nDNA networks are not included because the loci were invariable. Each circle indicates one haplotype, and its size is proportional to sampling frequency; black circles represent mutational steps when greater than one. An asterisk in *S*. *cyanea* network indicates Bahia haplotype (see text for detail). The black square represents an inferred but unsampled haplotype. (**b**) Bayesian inference based on the mtDNA data set. Colors indicate the four haplogroups. Posterior probability values: * ≥ 90%; ** ≥ 80%; *** ≥ 65%. (**c**) Measures of genetic differentiation (based on *Dxy*) compared between *S*. aff. *septentrionalis* and *S*. *cyanea* haplogroups for each locus. (**d**) Nucleotide diversity compared between *S. cyanea* and *S*. aff. *septentrionalis* for each locus.

The unrooted haplotype networks, BI, and AMOVA tests revealed contrasting degrees of phylogeographical structure between *S*. *cyanea* and *S*. aff. *septentrionalis* (Fig. 2, Tables S4 and S5). Despite the BI for *S*. *cyanea* exhibiting a lack of reciprocal monophyly, and hence no evidence for strong structuring, our unrooted haplotype network suggested a shallow split in the mtDNA-only haplogroups coincident with the neotectonic Guapiara lineament located in the southern state of São Paulo (e.g., Saadi *et al*.^68^; Thomé *et al*.^22^) (Figs 1, 2a and b). This result agrees with our cytogenetic data (see below). We named these haplogroups: (1) the Southern Guapiara lineament (SGL) haplogroup composed of specimens from Paraguay, Argentina and Brazilian states Rio Grande do Sul, Santa Catarina and Paraná; and (2) the Northern Guapiara Lineament (NGL) haplogroup which includes individuals from São Paulo, Minas Gerais and Espírito Santo states. The NGL and SGL haplogroups showed shallow divergence (based on *Dxy* and AMOVA test) in the mtDNA-only data set (12 S and EF1α were invariable) (see Fig. 2c, and Tables S4 and S5) as well as lack of evidence for reciprocal monophyly (see Fig. 2b). Furthermore, three individuals from Bahia, which are separated geographically from other members of the species, also formed a distinct haplotype and exhibited a different karyotype (see below), but due to the low sampling for DNA sequencing we focused on the NGL and SGL haplogroups and hence we excluded Bahia samples from subsequent analyses.

For *S*. aff. *septentrionalis*, two haplogroups based on both mtDNA and nDNA data sets were identified as follows: (1) the northern AF (NAF) haplogroup composed of individuals from Alagoas, Sergipe and Pernambuco states; and (2) the central and southern AF (CSAF) haplogroup composed of individuals from Bahia, Espírito Santo and São Paulo states. These haplogroups exhibited high degrees of differentiation in mtDNA but moderate differentiation in nDNA as reveled by *Dxy* and AMOVA (Tables S4 and S5). Also, the NAF is a paraphyletic group. Notably, the measures of genetic differentiation (based on *Dxy*) between *S*. aff. *septentrionalis* haplogroups are higher when compared with *S*. *cyanea* haplogroups; the only exception was that COI exhibited higher genetic differentiation in *S*. *cyanea* haplogroups (see Fig. 2c and Table S4).

Genetic diversity scores per locus and for each haplogroup are found in Table 1. The prediction that *S*. *cyanea* (southern AF) has lower genetic diversity than *S*. aff. *septentrionalis* (northern and central AF) was largely supported for all loci (approximately reduced by 3-fold in *S*. *cyanea*) (see Fig. 2d and Table 1). The only exception was that COI exhibited unexpectedly higher genetic diversity in *S*. *cyanea*. In *S*. aff. *septentrionalis*, the haplogroup NAF showed higher genetic diversity for all loci when compared to haplogoup CSAF, whereas *S*. *cyanea* did not exhibit a clear pattern regarding genetic diversity among its haplogroups.

### Historical demographic change

**Figure 3:**
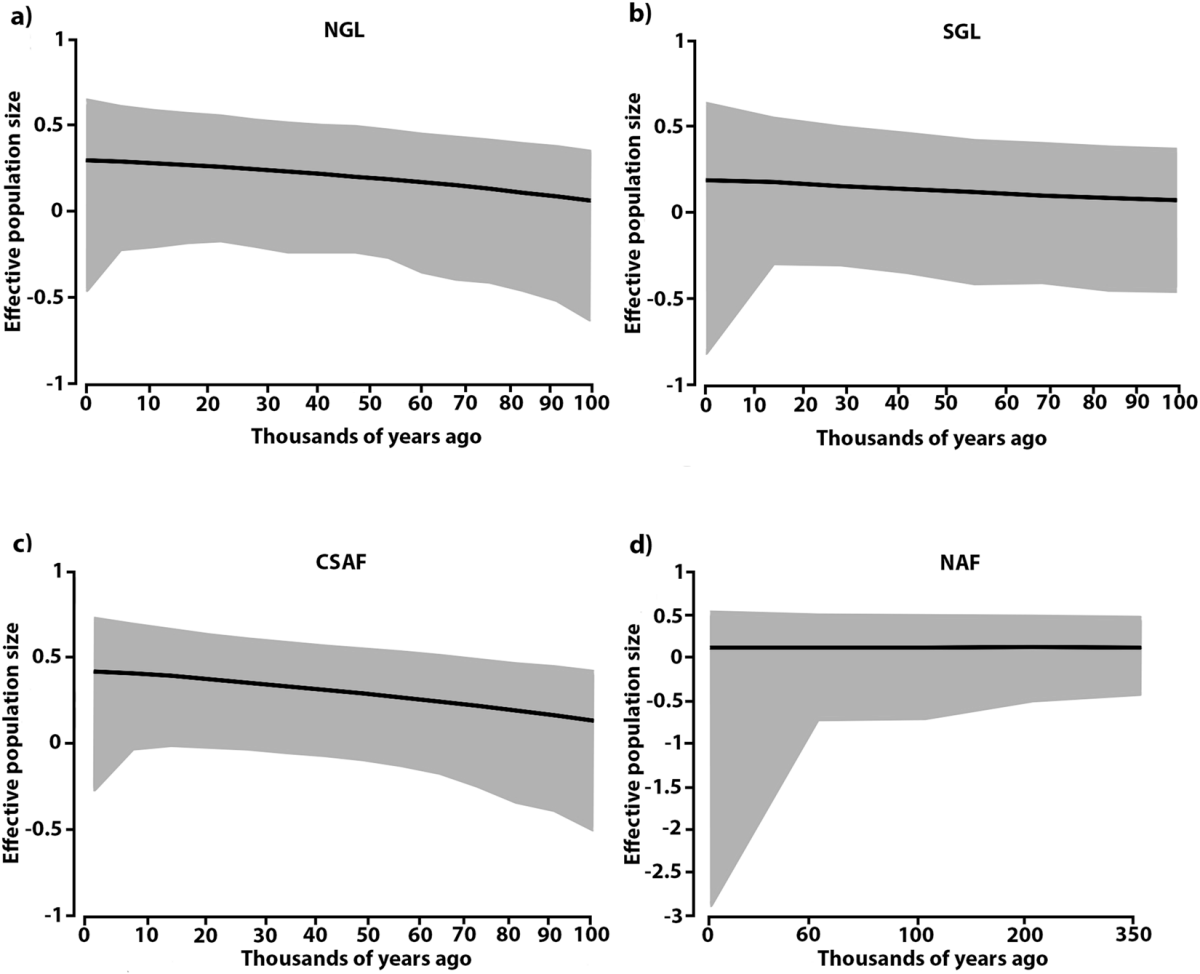
Comparison of demographic size through time. Extended Bayesian skyline plot (EBSP) using combined mtDNA and nDNA data sets of the major haplogroups of *S. cyanea* (**a)** and (**b**) and *S*. aff. *septentrionalis* (**c**) and (**d**). The EBSP shows median effective population sizes (black line) over the past and the shaded indicating the 95% HPD. The population size axis is on a logarithmic scale.

Neutrality tests (Table 1) capable of revealing signatures of population expansion exhibited significant departures from the standard neutral model in the following loci: *S*. *cyanea* haplogroup NGL – 16 S (Tajima’s *D*), CytB (Tajima’s *D*) and COI (Fu’s *Fs*); *S*. *cyanea* haplogroup SGL – CytB (Tajima’s *D*, Fu’s *Fs* and *R*_2_); and *S*. aff. *septentrionalis* haplogroup CSAF – CytB (Tajima’s *D*, Fu’s *Fs* and *R*_2_), COI (Tajima’s *D*, Fu’s *Fs* and *R*_2_), COII (Fu’s *Fs*), and CAD (Tajima’s *D*, Fu’s *Fs* and *R*_2_) (see Table 1). Our multilocus EBSP analysis showed a steady expansion of *S*. *cyanea* population size in the NGL (~2-fold increase), SGL (~1.5-fold increase) and *S*. aff. *septentrionalis* CSAF (~2-fold increase) from 100 kya to present (Fig. 3). There was no significant demographic change in *S*. aff. *septentrionalis* NAF during the past 100 kya (Fig. 3). Nevertheless, because confidence intervals (95% HPD) for all analyses are large and include values indicating negative population growth, we cannot dismiss the possibility of postglacial demographic retraction for NGL, SGL and CSAF, and preglacial demographic retraction for NAF.

### Suitable habitat in the present-day, LGM and LIG

**Figure 4:**
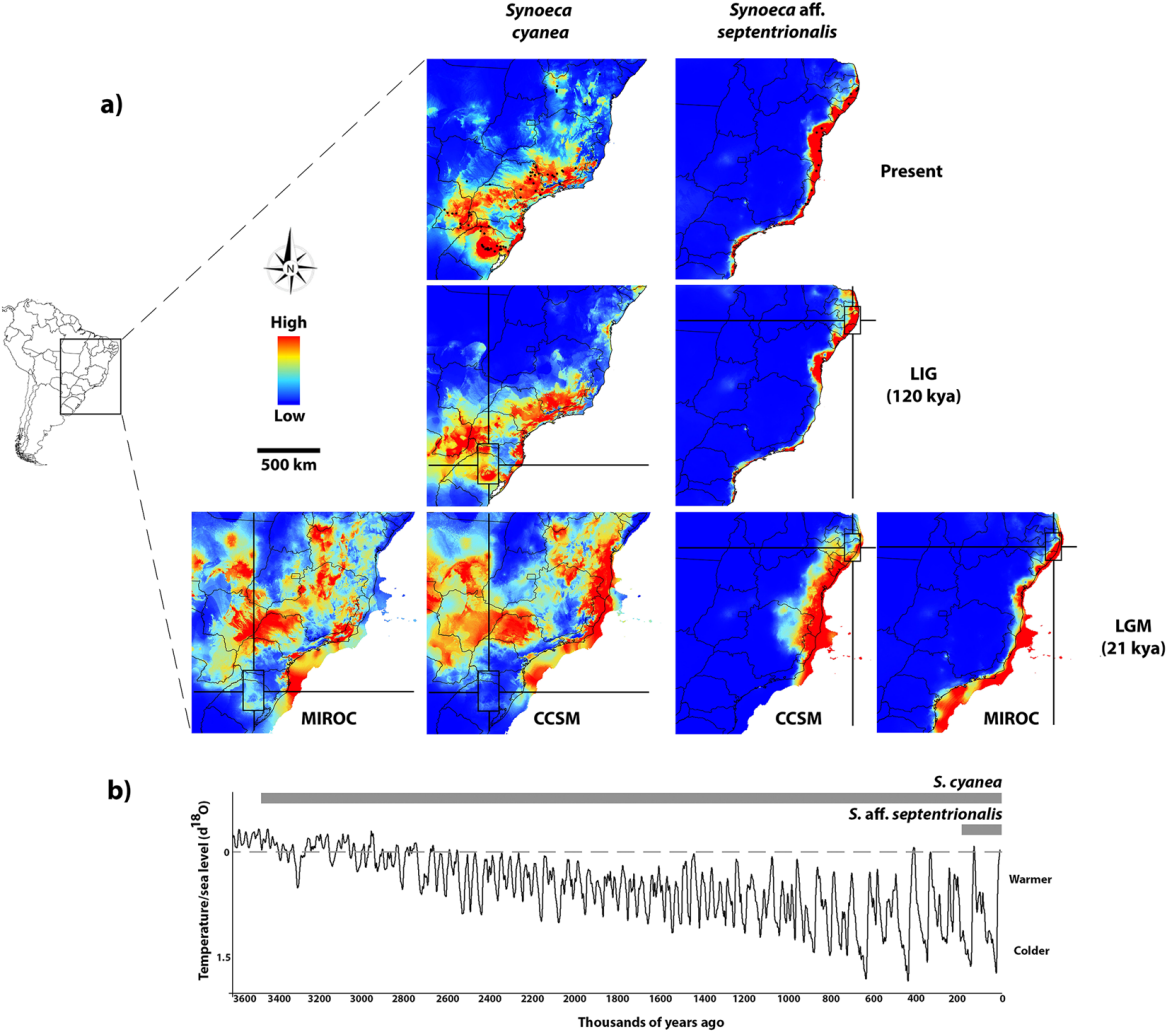
Test for range expansion, contraction or shift using ENM. (**a**) Present day, last glacial maximum (21,000 kya) and last interglacial (120–140 kya) ENMs for two ecologically distinctive *Synoeca* species. Color gradients show the relative climatic suitability scores and the rectangles represent the putative ancestral location (see also Fig. 5) for each wasp species. (**b**) Timeline representing the divergence time inferred for both *Synoeca* species (see text for details) and the Benthic δ^18^O records reproducing the historical fluctuations in the global temperature and sea level^96^. The map was generated using the software Quantum-GIS v2.8 (Open Source Geospatial Foundation Project, Beaverton, OR, USA) (http://www.qgis.org/en/site/forusers/download.html).

The predictive power of our ENM was higher than a random modeling for both species, as shown by average area under curve (AUC) values (*S*. *cyanea*, AUC_training_: 0.951 and AUC_test_: 0.910; *S*. aff. *septentrionalis*, AUC_training_: 0.992 and AUC_test_: 0.986) (Fig. 4a). Interestingly, irrespective of the climatic model used for LGM (MIROC or CCSM3), our ENMs predict a range shift for *S*. *cyanea* and an expansion for *S*. aff. *septentrionalis* during LGM (cold-dry) climate, but slight range contractions under LIG (warm-wet) and the present climate for both species. Results obtained under the MIROC model exhibited some differences when compared to the CCSM3 model, an expected result due to the different conditions simulated from these models (CCSM3 is derived from colder climatic conditions when compared to MIROC)^69^. ENMs for *S*. *cyanea* under LIG and current climatic conditions yielded similar reconstructions to each other and predicted highest climatic suitability compatible with the current distribution of this wasp. However, the LGM projection predicted that populations shift to lower elevations in two large areas, one in São Paulo, Mato Grosso, Mato Grosso do Sul states and Paraguay, and the other in the Espírito Santo, Rio de Janeiro and Bahia states, as well as into the exposed continental shelf. While the ENMs for *S*. aff. *septentrionalis* predicted higher climatic suitability in the Brazilian coastal lowlands under LIG (with slight range retraction) and the present climate, they also indicated range expansion into the continent and into the emerged continental shelf during LGM (see Fig. 4). Both MIROC and CCSM3 models predicted climatic suitability along the AF coast, with the MIROC model projecting additional climatic suitability in southern areas of the emerged continental shelf (Fig. 4).

### Contrasting historical movements

**Figure 5:**
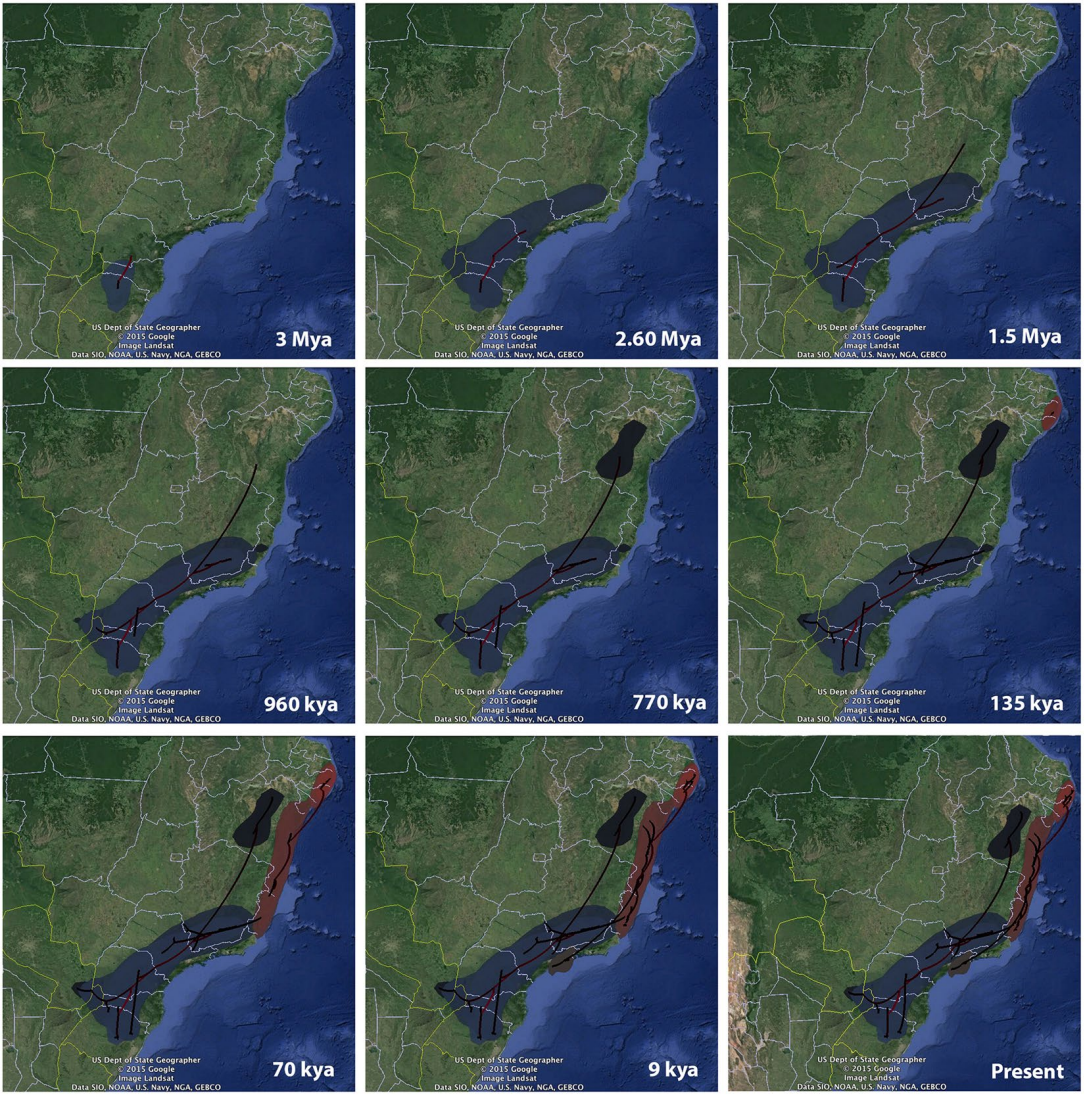
Bayesian reconstruction of the spatio-temporal dispersal of *S. cyanea* (blue shading) and *S*. aff. *septentrionalis* (red shading). Reconstructions are based on the maximum clade credibility tree estimated with a time-heterogeneous Relaxed Random Walk (RRW) model. Shading represents 80% HPD uncertainty in the location of ancestral branches with lighter and darker shades representing older and younger dispersal events, respectively. The map was generated using the software Google Earth 7.1.5.1557 (Google Inc. 2015) (https://www.google.com/earth/download/ge/agree.html).

Our Bayesian reconstruction of spatial-temporal movements (RRW model) suggested putative ancestral locations and colonization routes for *S*. *cyanea* and *S*. aff. *septentrionalis* (Fig. 5). The RRW model indicates the southern AF (more precisely, Rio Grande do Sul, Santa Catarina and southern Paraná states) as the putative ancestral location for *S*. *cyanea* and that this species experienced four main colonization routes: (1) this wasp began to spread to the north ~2.60 million years ago (mya), reaching São Paulo state; (2) around ~1.50 mya it reached Minas Gerais state; (3) around ~960 kya lineages from Rio Grande do Sul dispersed toward Misiones, Argentina; and (4) around ~770 kya lineages dispersed into Espírito Santo, Bahia and eastern Paraguay. For *S*. aff. *septentrionalis* the RRW model indicates the northern AF (precisely, Pernambuco and Alagoas states) as the putative ancestral location and infers two main colonization events: (1) an initial southern dispersal ~70 kya, reaching Bahia state, and (2) lineages reaching Espírito Santo and São Paulo states ~9 kya.

### Chromosome number cline and GC/AT-rich chromosomal segments

**Figure 6:**
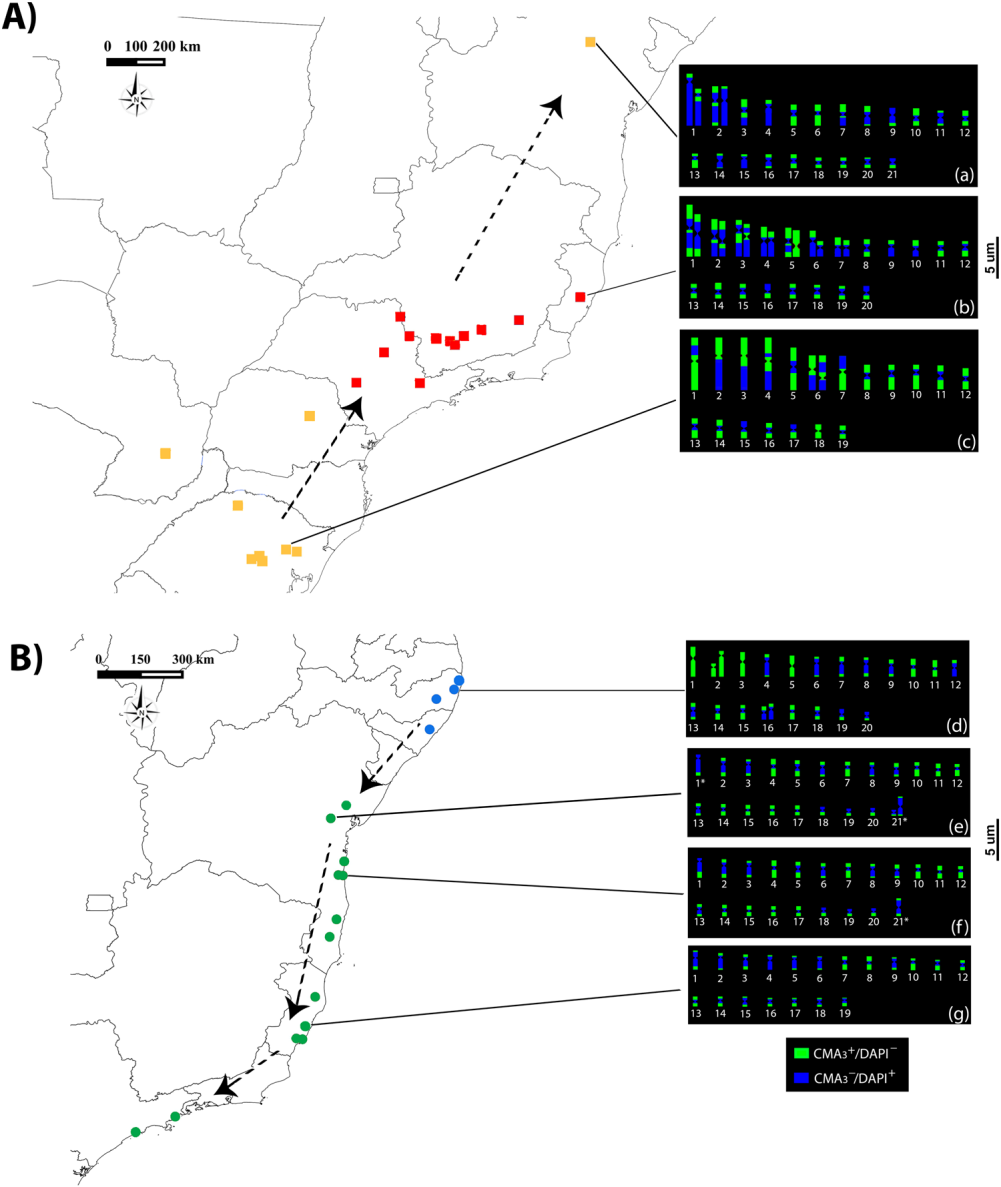
Spatial distribution of mtDNA haplotypes (see Fig. 2) and chromosomal cline witnessed for *S. cyanea* (**A**) and *S*. aff. *septentrionalis* (**B**). The small squares and circles represent haplotypes. Dashed arrows indicate the putative direction of historical dispersals (see text and Fig. 5 for details). Ideograms showing the CMA_3_/DAPI staining represent karyotype results (all information necessary to build the ideograms is shown in Figs S1 and S2). *S*. *cyanea*: (a) Senhor do Bonfim, BA, (b) Santa Teresa, ES, (c) Carlos Barbosa, RS; and *S*. aff. *septentrionalis*: (d) Moreno, PE, (e) Santa Teresinha, BA, (f) Ilhéus, BA, (g) Vila Regência, ES. An asterisk indicates specimens with the presence of only one chromosome of a homologous pair. The map was generated using the software Quantum-GIS v2.8 (Open Source Geospatial Foundation Project, Beaverton, OR, USA) (http://www.qgis.org/en/site/forusers/download.html).

Karyotypes from each target armadillo wasp species are shown in Fig. 6 (see also S1 and S2 for full details). Interestingly, both species exhibited variation in chromosome number in a latitudinal cline along the AF. *Synoeca cyanea* showed asymmetric karyotypes with large variation in chromosome size, representing three distinct karyotypes with the following chromosome numbers: 2n = 38 (Carlos Barbosa, RS; haplogroup SGL), 2n = 40 (Santa Teresa, ES; haplogroup NGL) and 2n = 42 (Senhor do Bonfim, Bahia haplotype) (Fig. 6A; for full details, see Table 2 and Supplementary Fig. S1). Additionally, karyotypes from Santa Teresa and Senhor do Bonfim showed size heteromorphism on the 1st through 7th pairs and 1st pair, respectively.

In *S*. aff. *septentrionalis*, we found symmetric karyotypes with a slight decreasing of chromosome size, representing three distinct karyotypes with the following chromosome numbers: 2n = 38 (Vila Regência, ES; haplogroup CSAF), 2n = 40 (Moreno, PE; haplogroup NAF) and 2n = 41 (Itabuna, BA; Santa Teresinha, BA; Itacaré, BA; and Ilhéus, BA; haplogroup CSAF) (Fig. 6B; for full details, see Table 2 and Supplementary Fig. S1). The karyotype from Moreno, PE showed size heteromorphism on the 2nd and 16th pairs. Moreover, we found specimens from CSAF with the presence of only one chromosome of a homologous pair [precisely the 1st (Santa Teresinha, BA) and 21th (Ilhéus, BA) pairs].

The CMA_3_/DAPI fluorochrome staining revealed distinct patterns regarding GC-rich sites (CMA_3_ positive) between the target species as well as among karyotypes. Depending on the chromosome, these sites were distributed in both chromosomal arms, in the terminal position, near the centromere, or throughout the entire chromosome (see Fig. 6 and Supplementary Fig. S2). Overall information regarding the specific chromosomal position of CMA_3_^+^ sites is outlined in Table 2. AT-rich sites (DAPI positive) were distributed in opposite position to CMA_3_^+^ sites, predominantly in the pericentromeric region.

## Discussion

The AF biome is characterized by high biological diversity^70^ and latitudinal topographic complexity. In our study, multiple lines of evidence allowed us to evaluate contrasting diversification processes proposed for the AF and generate insights into the microevolutionary history of two unusual model organisms in AF phylogeography, the armadillo wasp species *Synoeca cyanea* and *S*. aff. *septentrionalis*. Based on our results we propose that: (1) neotectonic and refugia played different roles for each species in shaping their respective genetic structures; (2) both species underwent demographic expansions even during the last glaciation; (3) they displayed contrasting patterns of spatial-temporal dispersal, and (4) they experienced substantial chromosomal changes associated with a latitudinal cline along the AF.

### Barriers and refugia shaped the genetic structure of AF social wasps

Our investigation into the population structure within *S*. *cyanea* and *S*. aff. *septentrionalis* revealed two major haplogroups in each species that are each distributed along a latitudinal gradient. For *S*. *cyanea* the main phylogeographic break (~2.60 mya) was coincident with the Guapiara lineament located in the southern São Paulo state (Figs 1 and 2), which separates the SGL haplogroup from the NGL haplogroup. This neotectonic barrier, active during the Quaternary^68^, has also been shown to have influenced spatially (although with temporal heterogeneity) the population genetic structure of the bee species *Melipona quadrifasciata* (0.23–0.84 mya)^71^, the snake species *Bothrops jararaca* (3.87 mya)^72^ and the toad species *Rhinella crucifer* (0.6 mya)^24^. A plausible explanation for why the genetic structure of *S*. *cyanea* has been influenced by the Guapiara lineament may be the strong association of this species with montane regions, especially in São Paulo and Paraná states (see Supplementary Table S3). The Guapiara lineament putatively created a deep valley (low altitude) that likely is not a suitable habitat for *S*. *cyanea* in this region, limiting gene flow between populations from the northern and southern parts of the Guapiara lineament. On the other hand, due to a sampling gap from northern Minas Gerais through Bahia, we were unable to locate precisely a putative phylogeographic break separating the Bahia haplotype from the other haplotypes.

For the lowland *S*. aff. *septentrionalis* our results specifically fit the scenario proposed by the CM model (refugia), as also observed for some other AF organisms^15, 20, 73^. Notably, this armadillo wasp species exhibited long-term persistence in areas of predicted refuges (e.g., Pernambuco and Bahia refuges) (see Fig. 4). *Synoeca* aff. *septentrionalis* also showed higher genetic diversity compared to *S*. *cyanea* (see Table 1, Figs 1 and 2), revealing expected differences between the two climatically different AF portions – northern (climatically stable) and southern (climatically unstable)^16^ – with no evidence of rivers or other physical barriers shaping its population structure. Moreover, *S*. aff. *septentrionalis* is younger than *S*. *cyanea*, with a divergence time (~180 kya, Menezes *et al*.^29^; here estimated in ~135 kya) that falls into the Pleistocene, a period marked by intense climatic swings^74, 75^. Together, this evidence suggests that the genetic structure of *S*. aff. *septentrionalis* populations was deeply influenced by climatic changes and consequently both Pernambuco and Bahia refuges could have served as a buffer to maintain its higher level of genetic diversity compared to *S*. *cyanea*.

Although we found strong evidence for a different primary role driving the genetic diversity of each AF species (a neotectonic barrier versus climate change), we cannot discard the possibility of a potential secondary role of these factors in shaping the genetic structure of AF biota. For example, we found unsuitable habitat during cooler periods (LGM) in the southern portion of the current distribution of *S*. *cyanea* (see Fig. 4a for location). It is possible that during glaciations, southern Brazil expanded its range of open areas (subtropical grasslands) to the north by at least 750 km (to latitude 20°S)^76, 77^. Thus, our paleomodeling supports a scenario of population migration from southern Brazil into lower elevations during glaciation with subsequent rapid post-glacial re-colonization. This scenario is congruent with our independent demographic analyses in that the NGL and SGL haplogroups showed moderate signals of population expansion (Table 1; Fig. 3). However, for *S*. aff. *septentrionalis* the Doce river may have played a secondary role as a barrier limiting gene flow, as we found populations separated by this river possessing two different karyotypes, specimens with 2n = 38 chromosomes (Vila Regência, ES) and specimens with 2n = 41 chromosomes (Itabuna, BA; Santa Teresinha, BA; Itacaré, BA; and Ilhéus, BA).

Phylogeographic structure in *S*. aff. *septentrionalis* is stronger than in *S*. *cyanea*, as indicated by comparison of *Dxy* and AMOVA values (Fig. 2c, Tables S4 and S5), reinforcing our idea of idiosyncratic histories and processes affecting their intraspecific genetic differentiation. It is possible that *S*. aff. *septentrionalis* individuals from the NAF and CSAF haplogroups were strongly affected by long-term separation from both Pernambuco and Bahia refuges, triggering the high to moderate levels of genetic differentiation found in both mtDNA and nDNA (mito-nuclear concordance) (see Fig. 2, Tables S4 and S5). In contrast, the shallow structure and low genetic diversity in *S*. *cyanea* can be explained by successive range shifts during glaciations (see Fig. 4), accompanied by bottleneck events that reduced genetic diversity, followed by rapid post-glacial re-colonization due to its resilience to cold climate. According to Arenas *et al*.^3^, range shifts can drastically reduce genetic diversity by recurrent founder effects. Moreover, a phylogeographic study on the bird species *Schiffornis virescens*, which has a distribution similar to *S*. *cyanea*, also showed shallow structure and low genetic diversity^78^. Additionally, the *S*. *cyanea* nDNA data set exhibited shallower signal of differentiation compared with mtDNA (mito-nuclear discordance), reflecting the intrinsic low mutation rate, large effective population size and differential retention of ancestral polymorphisms in nDNA data^79, 80^.

### Biological responses during the last glaciation

We compared the demographic history of two ecologically distinctive *Synoeca* species in order to evaluate the potential role of the Brazilian emerged continental shelf during the LGM as proposed by the Atlantis hypothesis. Based on our tests of demographic change (Table 1, Figs 3 and 4), these armadillo wasps underwent demographic expansion even during the LGM. Despite *S*. *cyanea* experiencing a range shift during glaciation (see Fig. 4), this species showed a steady expansion in population size for both NGL and SGL haplogroups throughout the last 100 kya (Fig. 3). One plausible explanation for this demographic expansion is that the shift from a panmictic population to multiple subdivided populations (i.e., dispersed LGM refugia or range disjunction) could cause an increase in effective population size without a considerable change in population size (abundance)^13^; alternatively, the intrinsic tolerance to colder climates could shield AF montane biota during glaciations^23^.

There is growing evidence that, indeed, the northern portion of the AF served as refugia to lowland organisms^14, 15^. Moreover, Leite *et al*.^26^ used a combination of paleomodeling and coalescent simulations on five mammal species to propose that the emerged Brazilian continental shelf experienced a favorable climate allowing forests and forest-dwelling species to expand during the LGM. Notably, the forest-dwelling *S*. aff. *septentrionalis* fits well with the expectations proposed by refugia and Atlantis Forest hypotheses for the AF, as reveled by our phylogeographic analyses (Table 1, Fig. 3) and paleomodeling (Fig. 4). Glacial-interglacial cycles played a crucial role in eustatic changes, and during LGM global sea levels dropped to approximately −120 m, whereas the rate of sea-level rise reached at least 1.2 m per century during deglaciation^81^. Assuming that an exposed Brazilian continental shelf during glaciation was forested, this available area could have been favorable to colonization by *S*. aff. *septentrionalis* populations, and may have facilitated the migration of this species south across the latitudinal gradient. This hypothesis is congruent with the demographic expansion observed for CSAF but not for NAF (the latter is probably due to reduced emerged available area; see Table 1, Figs 3 and 4), as well as the absence of population structure among Bahia, Espírito Santo and São Paulo populations. A similar pattern of demographic expansion and shallow population structure, probably influenced by eustatic change, was witnessed in another hymenopteran, the sand dune ant *Mycetophylax simplex*, distributed along the southern portion of the AF coast^82^. Hence, we argue that the emerged Brazilian continental shelf may have had a significant role in influencing demographic expansion^26^ and gene flow in AF biota, rather than acting as putative barrier as proposed by Leite *et al*.^26^.

### Using Bayesian phylogeography and ENM to infer geographic origin

Reconstructions of historical dispersal using the RRW model recently have been applied to infer the dispersal dynamics of multiple organisms including lizards^32, 83^, salamanders^84^, moles^85^ and ants^86^. Our RRW model revealed contrasting patterns of historical movements along the AF between the two armadillo wasps. *Synoeca cyanea* was inferred to have migrated from south to north, whereas *S*. aff. *septentrionalis* spread from north to south (Fig. 5), a putative scenario also supported by ENM (Fig. 4) and cytogenetic data (see next section). Critically, our RRW model requires further validation using fine-scale sampling and structured coalescent methods that are expected to be less affected by sampling biases^87^. Nevertheless, we note that our RRW models are concordant with our independent paleoclimatic reconstructions. The divergence time inferred for *S*. *cyanea* falls during the Pliocene [herein ~3 mya; in a previous study^29^ ~3.46 mya], a period when global temperatures were relatively warm and similar to the present climate (see Fig. 4b)^72, 73^. Thus, the inferred ancestral location for *S*. *cyanea* in southern AF was climatically suitable during its putative divergence time. The same idea can be applied to *S*. aff. *septentrionalis* that arose during the penultimate glacial period [herein ~135 kya; in a previous study^29^ ~180 kya], specifically during marine isotope stage 6 (MIS6) (~130–185 kya) (see Fig. 4b)^74, 75, 88^. Thus, the inferred ancestral location for *S*. aff. *septentrionalis* in northern AF also was climatically suitable during its putative divergence time.

### Cytogenetics meets phylogeography

Phylogenetic analysis is a cornerstone in molecular and evolutionary biology studies. Hence, our cytogenetic interpretations benefit from the insights provided by our Bayesian phylogeographic analyses, allowing us to more accurately infer the chromosomal microevolutionary history of these armadillo wasps. Because we found no evidence of cryptic species (based on our molecular markers and the absence of morphological differences), these insects showed remarkable intraspecific variation involving chromosome number and GC/AT-rich segments along their geographic range. While *S*. *cyanea* nDNA is organized into large and small chromosomes, *S*. aff. *septentrionalis* nDNA is organized mostly into small chromosomes, implying accumulation of chromosomal rearrangements (e.g., inversions, fissions and/or fusions) during the chromosomal evolutionary history of *Synoeca*. Additionally, the intrapopulational chromosome heteromorphism witnessed in these insects is additional evidence of considerable amount of chromosomal changes in their karyotypes. Considering our phylogeographic analyses for these species (Fig. 5), as discussed above, and their intraspecific karyotypic variability (Table 2, Figs 6, S1 and S2), we have strong indication that, indeed, these chromosomal changes and the direction of population dispersal were latitudinally oriented along the AF.

Integrating cytogenetic and phylogeographic analyses also revealed two intriguing features about GC content (here as CMA_3_^+^ sites) within these *Synoeca* species: (1) GC content decreased during their chromosomal microevolutionary history; and (2) change in the chromosomal distribution of GC content seems to be more dynamic in *S*. *cyanea*, especially between pericentromeric regions (Fig. 6). It is not surprising that heterochromatic regions composed of repetitive DNA are hotspots for chromosomal rearrangements^89–91^. Although we did not perform cytogenetic tests to detect heterochromatic regions and/or specific families of repetitive DNA, it is possible that GC-rich sites in *Synoeca* represent these types of genomic regions, as verified in other insects including beetles^92^ and hymenopterans including solitary^93^ and social^91^ wasps.

Chromosomal Robertsonian fusions and fissions seem to have played a significant role in the intense reshuffling of *Synoeca* karyotypes. Notably, these rearrangements are common evolutionary changes that have been reported for multiples species^94^, and seem to be frequent during the chromosomal evolutionary history of swarm-founding social wasps^91, 95^. These chromosomal rearrangements can produce karyotype asymmetry, chromosome number change and loss of chromosomal segments, as well as producing occasionally post-zygotic reproductive barriers, limiting gene flow^33, 34, 89^. Assuming that chromosomal rearrangements are frequent in social wasps and that chromosomal changes can accumulate within isolated populations, karyotypic differences, barriers and refugia could together reduce gene flow over time among *Synoeca* populations, triggering the phylogeographic patterns here described.

## Electronic supplementary material

Supplemental materials
