# Supplementary material for: The roles of barriers, refugia, and chromosomal clines underlying diversification in Atlantic Forest social wasps
Source: Sci Rep. 2017 Aug 9;7:7689. doi: 10.1038/s41598-017-07776-7 (PMC5550474; doi:10.1038/s41598-017-07776-7)

Supplemental Tables and Figures: The roles of barriers, refugia, and chromosomal clines underlying diversification in Atlantic Forest social wasps. **Scientific Reports**.

Rodolpho S. T. Menezes, Seán G. Brady, Antônio F. Carvalho, Marco A. Del Lama and Marco A. Costa

Table S1: Geographical information for all samples included in this study and GenBank accession reference.

Synoeca cyanea				**GenBank accession number**						
ID/Locality	Longitude	Latitude	H	16S	12S	COI	COII	CytB	CAD	EF1α
C1. Senhor do Bonfim, BA, Brazil	-40.2231	-10.4458	Bahia	KX941503	KX941536	KX941557	KX941583	KX941605	KX941640	KX941662
C2. Senhor do Bonfim, BA, Brazil	-40.2231	-10.4458	Bahia	KX941504	KX941537	KX941558	KX941584	KX941606	KX941641	KX941663
C3. Rio de Contas, BA, Brazil	-41.8169	-13.7345	Bahia	KX941505	-	KX941559	-	KX941607	-	-
C4. Santa Teresa, ES, Brazil	-40.5995	-19.9228	NGL	KX941506	-	KX941560	KX941585	KX941608	KX941642	KX941664
C5. Viçosa, MG, Brazil	-42.875	-20.7748	NGL	KX941507	KX941538	KX941561	KX941586	KX941609	KX941643	KX941665
C6. Viçosa, MG, Brazil	-42.875	-20.7748	NGL	KX941508	KX941539	KX941562	KX941587	KX941610	KX941644	KX941666
C7. Viçosa, MG, Brazil	-42.875	-20.7748	NGL	KX941509	KX941540	KX941563	KX941588	KX941611	KX941645	KX941667
C8. Viçosa, MG, Brazil	-42.875	-20.7748	NGL	KX941510	KX941541	KX941564	KX941589	KX941612	KX941646	KX941668
C9. Viçosa, MG, Brazil	-42.875	-20.7748	NGL	KX941511	-	KX941565	KX941590	KX941613	KX941647	KX941669
C10. Varginha, MG, Brazil	-45.4303	-21.5514	NGL	KX941512	KX941542	KX941566	KX941591	KX941614	KX941648	KX941670
C11. Varginha, MG, Brazil	-45.4303	-21.5514	NGL	KX941513	KX941543	KX941567	KX941592	KX941615	KX941649	KX941671
C12. Três Corações, MG, Brazil	-45.2533	-21.6969	NGL	KX941514	KX941544	KX941568	KX941593	KX941616	KX941650	KX941672
C13. Arceburgo, MG, Brazil	-46.94	-21.3642	NGL	KX941515	KX941545	KX941569	KX941594	KX941617	KX941651	KX941673
C14. São João Del Rei, MG, Brazil	-44.2617	-21.1356	NGL	KX941516	KX941546	KX941570	-	KX941618	KX941652	KX941674
C15. Alfenas, MG, Brazil	-45.9462	-21.4637	NGL	KX941517	KX941547	KX941571	KX941595	KX941619	KX941653	KX941675
C16. Ingaí, MG, Brazil	-44.9258	-21.361	NGL	KX949576	-	-	-	KX941620	-	-
C17. Ingaí, MG, Brazil	-44.9258	-21.361	NGL	KX949577	-	-	-	-	-	-
C18. Telêmaco Borba, PR, Brazil	-50.6423	-24.3378	SGL	KX941518	-	-	KX941596	KX941621	KX941654	-
C19. Telêmaco Borba, PR, Brazil	-50.6423	-24.3378	SGL	KX941519	KX941548	-	KX941597	KX941622	-	-
C20. Iraí, RS, Brazil	-53.2506	-27.1936	SGL	KX941520	-	-	-	KX941623	-	-
C21. Nova Petrópolis, RS, Brazil	-51.1252	-29.3748	SGL	KX941521	KX941549	KX941573	KX941598	KX941624	KX941655	KX941676
C22. Boa Vista das Missões, RS, Brazil	-53.3142	-27.6631	SGL	KX941522	-	-	-	KX941625	-	-
C23. Candelária, RS, Brazil	-52.7889	-29.6692	SGL	KX941523	-	KX941574	-	KX941626	-	-
C24. Sinimbu, RS, Brazil	-52.5217	-29.5386	SGL	KX941524	-	KX941575	-	KX941627	-	-
C25. Santa Cruz do Sul, RS, Brazil	-52.4258	-29.7175	SGL	KX941525	KX941550	-	-	KX941628	-	-
C26. Santa Cruz do Sul, RS, Brazil	-52.4258	-29.7175	SGL	KX941526	-	-	-	KX941629	-	-
C27. Santa Cruz do Sul, RS, Brazil	-52.4258	-29.7175	SGL	KX941527	-	-	-	KX941630	-	-
C28. Carlos Barbosa, RS, Brazil	-51.5036	-29.2975	SGL	KX941528	KX941551	KX941576	KX941599	KX941631	KX941656	KX941677
C29. *Nova Teutônia, SC, Brazil	-52.4162	-27.1611	SGL	-	-	KX941577	-	-	-	-

C30. São Carlos, SP, Brazil	-47.8825	-21.9759	NGL	KX941529	KX941552	KX941578	KX941600	KX941632	KX941657	KX941678
C31. São Carlos, SP, Brazil	-47.8825	-21.9759	NGL	KX941530	KX941553	KX941579	KX941601	KX941633	KX941658	KX941679
C32. São Carlos, SP, Brazil	-47.8825	-21.9759	NGL	KX941531	KX941554	KX941580	KX941602	KX941634	KX941659	KX941680
C33. Atibaia, SP, Brazil	-46.5503	-23.1169	NGL	KX941532	KX941555	KX941581	KX941603	KX941635	KX941660	KX941681
C34. Avaré, SP, Brazil	-48.9096	-23.0987	NGL	KX941533	KX941556	KX941582	KX941604	KX941636	KX941661	KX941682
C35. Patrocínio Paulista, SP, Brazil	-47.278	-20.6509	NGL	KX941534	-	-	-	KX941637	-	-
C36. *Paso Yobai, Guairá, Paraguay	-55.9968	-25.7266	SGL	KX941535	-	-	-	KX941638	-	-
C37. Wanda, Misiones, Argentina	-54.5829	-25.9822	SGL	-	-	-	-	KX941639	-	-
Synoeca* aff. *septentrionalis				**GenBank accession number**						
Locality	Longitude	Latitude	H	16S	12S	COI	COII	CytB	CAD	EF1α
I1. Pilar, AL, Brazil	-35.9658	-9.5605	NAF	KX941683	-	KX941723	KX941746	KX941767	KX941790	KX941811
I2. Santa Teresinha, BA, Brazil	-39.5102	-12.7522	CSAF	KX941684	KX941707	KX941724	KX941747	KX941768	KX941791	KX941812
I3. Itabuna, BA, Brazil	-39.239	-14.7825	CSAF	KX941685	KX941708	KX941725	KX941748	KX941769	KX941792	KX941813
I4. Feira de Santana, BA, Brazil	-38.9553	-12.2823	CSAF	KX941686	-	KX941726	KX941749	KX941770	KX941793	KX941814
I5. Itacaré, BA, Brazil	-39.0284	-14.2933	CSAF	KX941687	KX941709	KX941727	KX941750	KX941771	KX941794	KX941815
I6. Itamaraju, BA, Brazil	-39.5501	-17.0007	CSAF	KX941688	KX941710	KX941728	KX941751	KX941772	KX941795	KX941816
I7. Itamaraju, BA, Brazil	-39.5501	-17.0007	CSAF	KX941689	KX941711	KX941729	KX941752	KX941773	KX941796	KX941817
I8. Porto Seguro, BA, Brazil	-39.3051	-16.3732	CSAF	KX941690	KX941712	KX941730	KX941753	KX941774	KX941797	KX941818
I9. Porto Seguro, BA, Brazil	-39.3051	-16.3732	CSAF	KX941691	KX941713	KX941731	KX941754	KX941775	KX941798	KX941819
I10. Ilhéus, BA, Brazil	-39.0751	-14.8005	CSAF	KX941692	KX941714	KX941732	KX941755	KX941776	KX941799	KX941820
I11. Ilhéus, BA, Brazil	-39.0751	-14.8005	CSAF	KX941693	KX941715	KX941733	KX941756	KX941777	KX941800	KX941821
I12. Alfredo Chaves, ES, Brazil	-40.7463	-20.6433	CSAF	KX941694	-	KX941734	KX941757	KX941778	KX941801	KX941822
I13. Linhares, ES, Brazil	-40.0694	-19.1514	CSAF	KX941695	KX941716	KX941735	KX941758	KX941779	KX941802	KX941823
I14. Linhares, ES, Brazil	-40.0694	-19.1514	CSAF	KX941696	KX941717	KX941736	KX941759	KX941780	KX941803	KX941824
I15. Linhares, ES, Brazil	-40.0694	-19.1514	CSAF	KX941697	KX941718	KX941737	KX941760	KX941781	KX941804	KX941825
I16. Vila Regência, ES, Brazil	-40.4273	-20.2057	CSAF	KX941698	KX941719	KX941738	KX941761	KX941782	KX941805	KX941826
I17. Vila Regência, ES, Brazil	-40.4273	-20.2057	CSAF	KX941699	KX941720	KX941739	KX941762	KX941783	KX941806	KX941827
I18. *Guarapari, ES, Brazil	-40.5246	-20.6711	CSAF	KX941700	-	-	-	KX941784	-	-
I19. Igarassu, PE, Brazil	-34.9084	-7.8085	NAF	KX941701	-	KX941740	KX941763	KX941785	KX941807	KX941828
I20. Igarassu, PE, Brazil	-34.9084	-7.8085	NAF	KX941702	KX941721	KX941741	KX941764	KX941786	KX941808	KX941829
I21. Moreno, PE, Brazil	-35.0884	-8.1276	NAF	KX941703	KX941722	KX941742	KX941765	KX941787	KX941809	-
I22. *Bonito, PE, Brazil	-35.7257	-8.4725	NAF	KX941704	-	KX941743	-	-	-	-
I23. Ubatuba, SP, Brazil	-45.0867	-23.4444	CSAF	KX941705	-	KX941744	KX941766	KX941788	KX941810	KX941830
I24. *Praia Grande, SP, Brazil	-46.5077	-24.0027	CSAF	KX941706	-	KX941745	-	KX941789	-	-

H: haplogroups and Bahia haplotype (see text for details); * museum specimens (≤ 50 years) from USNM Entomology (Smithsonian Institution).

Table S2: Descriptive list of primers used in this study.

Primer	Primer sequence (5´-3´)	PCR temp	Reference*
**16S**		94 ºC 1 min, 47 ºC 1 min and 30 s, 64 ºC 1 min and 30 s - 40 cycles	
LR13943F	CACCTGTTTATCAAAAACAT		Costa et al. (2003)
LR13392R	CGTCGATTTGAACTCAAATC		Costa et al. (2003)
**12S**		94 ºC 30 s, 43 ºC 45 s, 72 ºC 45 s - 40 cycles	
12S ai (SR-J-14233)	AAACTAGGATTAGATACCCTATTAT		Simon et al. (1994)
12S bi (SR-N-14588)	AAGAGCGACGGGCGATGTGT		Simon et al. (1994)
**COI**		95 ºC 30 s, 45 ºC 1min, 72 ºC 1 min - 35 cycles	
CI-J-1718	GGAGGATTTGGAAATTGATTAGTTCC		Simon et al. (1994)
CI-N-2191	GGTAAAATTAAAATATAAACTTC		Kambhampati and Smith (1995)
**COII**		94 ºC 30 s, 50 ºC 45 s, 72 ºC 45 s - 40 cycles	
E2	GGCAGAATAAGTGCATTG		Garnery et al. (1992)
COII1-2	ATTTTATACCACAAATTTCTGAACATTG		Saito and Kojima (2011)
**CytB**		94 ºC 30 s, 54 ºC 20 s, 70 ºC 1 min - 40 cycles	
CRO91A	TATGTACTACCATGAGGACAAATATC		Crozier et al. (1991)
CRO91B	ATTACACCTCCTAATTTATTAGGAAT		Crozier et al. (1991)
**CAD**		94 ºC 1 min, 52 ºC 1 min, 72 ºC 1 min and 30 s - 35 cycles	
apCADfor4	TGGAARGARGTBGARTACGARGTGGTYCG		Danforth et al. (2006)
apCADrev4a	GGCCAYTGNGCNGCCACYGTGTCTATYTGYTTNACC		Danforth et al. (2006)
**EF1α**		94 ºC 1 min, 54 ºC 1 min, 72 ºC 1 min and 30 s - 35 cycles	
HaF2For1	GGGYAAAGGWTCCTTCAARTATGC		Danforth and Ji (1998)
F2-rev1	AATCAGCAGCACCTTTAGGTGG		Danforth and Ji (1998)

*Reference:

Costa MA, Del Lama MA, Melo GAR, Sheppard WS (2003) Molecular phylogeny of the stingless bees (Apidae, Apinae, Meliponini) inferred from mitochondrial 16S rDNA sequences. *Apidologie*, **34**, 73-84.

Crozier RH, Crozier YC (1993) The mitochondrial genome of the honeybee *Apis mellifera*: complete sequence and genome organization. *Genetics*, **133**, 97-117.

Danforth BN, Fang J, Sipes SD (2006) Analysis of family level relationships in bees (Hymenoptera: Apiformes) using 28S and two previously unexplored nuclear genes: CAD and RNA polymerase II. *Molecular Phylogenetics and Evolution*, **39**, 358-372.

Danforth BN, Ji S (1998) Elongation factor-1 alpha occurs as two copies in bees: implications for phylogenetic analysis of EF-1 alpha sequences in insects. *Molecular Biology and Evolution*, **15**, 225-235.

Garnery L, Cornuet JM, Solignac M (1992) Evolutionary history of the honey bee *Apis mellifera* inferred from mitochondrial DNA analysis. *Molecular Ecology*, **1**, 145-154.

Kambhampati S, Smith PT (1995) PCR primers for the amplification of four insect mitochondrial gene fragments. *Insect Molecular Biology*, **4**, 233-236.

Saito F, Kojima J (2011) Phylogenetic analysis and biogeography of the nocturnal hornets, *Provespa* (Insecta: Hymenoptera: Vespidae: Vespinae). *Species Diversity*, **16**, 65-74.

Simon C, Frati F, Bechenbach A, *et al*. (1994) Evolution, weighting, and phylogenetic utility of mitochondrial gene sequence and compilation of conserved polymerase chain reaction primers. Annals of Entomological Society of America, **87**, 651-701.

Table S3: Georeferenced species presence data points used for Ecological niche modeling (ENM).

Synoeca cyanea				
Locality	Longitude	Latitude	Altitude	Source
Iguazú, Argentina	-54.35	-25.8471	235m	AMNH
Wanda, Misiones, Argentina	-54.5829	-25.9822	181m	Donation
Loreto, Misiones, Argentina	-55.5315	-27.3161	113m	USNM; SI
Dos de Mayo, Misiones, Argentina	-54.6501	-27.0333	542m	AMNH
Puerto Iguazú, Misiones, Argentina	-54.5824	-25.595	148m	AMNH
Barreiras, Bahia, Brazil	-44.9767	-12.1797	559m	Santos et al. 2009
São Desidério, Bahia, Brazil	-44.9706	-12.3694	572m	Santos et al. 2009
Senhor do Bonfim, Bahia, Brazil	-40.2231	-10.4458	840m	Surveying
Riachão das Neves, Bahia, Brazil	-44.8979	-11.7517	450m	Santos et al. 2009
Rio de Contas, Bahia, Brazil	-41.8169	-13.7345	482m	Donation
Santa Teresa, Espírito Santo, Brazil	-40.5995	-19.9228	762m	Surveying
Dourados, Mato Grosso do Sul, Brazil	-54.8247	-22.2074	431m	Donation
Viçosa, Minas Gerais, Brazil	-42.875	-20.7748	695m	Surveying
Varginha, Minas Gerais, Brazil	-45.4303	-21.5514	892m	Surveying
Ritápolis, Minas Gerais, Brazil	-44.3627	-21.0207	1003m	J.M.F. Camargo (RPSP)
Três corações, Minas Gerais, Brazil	-45.2533	-21.6969	882m	Surveying
Arceburgo, Minas Gerais, Brazil	-46.94	-21.3642	717m	Surveying
São João Del Rei, Minas Gerais, Brazil	-44.2617	-21.1356	931m	Surveying
Alfenas, Minas Gerais Brazil	-45.9462	-21.4637	901m	Surveying
Ingaí, Minas Gerais, Brazil	-44.9258	-21.361	911m	Donation
Telêmaco Borba, Paraná, Brazil	-50.6423	-24.3378	797m	Donation
Ponta Grossa, Paraná, Brazil	-50.0241	-25.2455	798m	AMNH
Itatiaia, Rio de Janeiro, Brazil	-44.5797	-22.4907	414m	MZUSP
Nova Petrópolis, Rio Grande do Sul, Brazil	-51.1252	-29.3748	527m	Donation
Boa vista das missões, Rio Grande do Sul, Brazil	-53.3142	-27.6631	563m	Donation
Candelária, Rio Grande do Sul, Brazil	-52.8046	-29.6482	147m	Donation
Sinimbu, Rio Grande do Sul, Brazil	-52.5153	-29.5333	111m	Donation
Santa Cruz do Sul, Rio Grande do Sul, Brazil	-52.4094	-29.7131	174m	Donation
Carlos Barbosa, Rio Grande do Sul, Brazil	-51.5201	-29.2935	632m	Donation
Osório, Rio Grande do Sul, Brazil	-50.2751	-29.8802	114m	Andena et al. 2009
Arroio do Tigre, Rio Grande do Sul, Brazil	-53.0941	-29.3346	406m	Somavilla et al. 2010
Campo Bom, Rio Grande do Sul, Brazil	-51.0451	-29.6793	33m	Somavilla et al. 2010
Canela, Rio Grande do Sul, Brazil	-50.8133	-29.3804	821m	Somavilla et al. 2010
Estrela Velha, Rio Grande do Sul, Brazil	-53.1646	-29.1851	380m	Somavilla et al. 2010
General Câmara, Rio Grande do Sul, Brazil	-51.7677	-29.9014	27m	Somavilla et al. 2010
Novo Hamburgo, Rio Grande do Sul, Brazil	-51.179	-29.7034	68m	Somavilla et al. 2010

São Francisco de Paula, Rio Grande do Sul, Brazil	-50.5665	-29.4527	875m	Somavilla et al. 2010
Sarandi, Rio Grande do Sul, Brazil	-52.9086	-27.9381	553m	Somavilla et al. 2010
Sobradinho, Rio Grande do Sul, Brazil	-53.0083	-29.4174	497m	Somavilla et al. 2010
Vale do Sol, Rio Grande do Sul, Brazil	-52.6899	-29.6066	76m	Somavilla et al. 2010
Venâncio Aires, Rio Grande do Sul, Brazil	-52.179	-29.5981	36m	Somavilla et al. 2010
Vera Cruz, Rio Grande do Sul, Brazil	-52.5425	-29.7149	54m	Somavilla et al. 2010
Iraí, Rio Grande do Sul, Brazil	-53.2506	-27.1936	215m	Donation
Nova Teutônia, Santa Catarina, Brazil	-52.4162	-27.1611	352m	USNM; SI
São José, Santa Catarina, Brazil	-48.7791	-27.5801	475m	AMNH
Blumenau, Santa Catarina, Brazil	-49.0889	-26.9263	132m	AMNH
São Carlos, São Paulo, Brazil	-47.8825	-21.9759	876m	Surveying
Atibaia, São Paulo, Brazil	-46.5503	-23.1169	818m	Surveying
Avaré, São Paulo, Brazil	-48.9096	-23.0987	773m	Surveying
Teodoro Sampaio, São Paulo, Brazil	-52.3164	-22.5501	361m	J.M.F. Camargo (RPSP)
Luiz Antônio, São Paulo, Brazil	-47.8168	-21.6128	534m	J.M.F. Camargo (RPSP)
Rio Claro, São Paulo, Brazil	-47.5075	-22.4214	702m	J.M.F. Camargo (RPSP)
Serrana, São Paulo, Brazil	-47.6104	-21.2205	643m	J.M.F. Camargo (RPSP)
Ribeirão Preto, São Paulo, Brazil	-47.8476	-21.2179	601m	J.M.F. Camargo (RPSP)
Patrocínio Paulista, São Paulo, Brazil	-47.278	-20.6509	763m	Donation
Santo Amaro, São Paulo, Brazil	-46.7355	-23.7063	770m	USNM; SI
Itapeva, São Paulo, Brazil	-48.9326	-23.9679	688m	AMNH
Itú, São Paulo, Brazil	-47.2702	-23.2575	603m	MZUSP
Pedregulho, São Paulo, Brazil	-47.4823	-20.2619	1007m	Andena et al. 2009
Santa Rosa de Viterbo, São Paulo, Brazil	-47.3506	-21.5073	748m	Andena et al. 2009
San Bernardino, Cordillera, Paraguay	-57.2863	-25.3146	88m	USNM; SI
Puerto Bertoni, Paraguay	-54.594	-25.6563	203m	USNM; SI
Paso Yobai, Guairá, Paraguay	-55.9968	-25.7266	214m	USNM; SI
Sapucai, Paraguarí, Paraguay	-56.9566	-25.6688	170m	USNM; SI
Villarica, Guairá, Paraguay	-56.4294	-25.7465	146m	USNM; SI
Pirapó, Itapuá, Paraguay	-55.5467	-26.861	139m	AMNH
Synoeca* aff. *septentrionalis				
Locality	Longitude	Latitude	Altitude	Source
Pilar, Alagoas, Brazil	-35.9658	-9.5605	123m	Surveying
Santa Teresinha, Bahia, Brazil	-39.5102	-12.7522	199m	Surveying
Itabuna, Bahia, Brazil	-39.239	-14.7825	48m	Surveying
Feira de Santana, Bahia, Brazil	-38.9553	-12.2823	240m	Donation
Itacaré, Bahia, Brazil	-39.0284	-14.2933	91m	Surveying
Itamaraju, Bahia, Brazil	-39.5501	-17.0007	106m	Surveying
Porto Seguro, Bahia, Brazil	-39.3051	-16.3732	105m	Surveying

Ilhéus, Bahia, Brazil	-39.0751	-14.8005	10m	Surveying
Alfredo Chaves, Espírito Santo, Brazil	-40.7463	-20.6433	19m	Surveying
Linhares, Espírito Santo, Brazil	-40.0694	-19.1514	65m	Surveying
Vila Regência, Espírito Santo, Brazil	-40.4273	-20.2057	11m	Surveying
Guarapari, Espírito Santo, Brazil	-40.5246	-20.6711	16m	USNM; SI
Igarassu, Pernambuco, Brazil	-34.9084	-7.8085	21m	Surveying
Moreno, Pernambuco, Brazil	-35.0884	-8.1276	117m	Surveying
Bonito, Pernambuco, Brazil	-35.7257	-8.4725	446m	USNM; SI
Recife, Pernambuco, Brazil	-34.9475	-8.01	11m	MZUSP
Rio de Janeiro, Rio de Janeiro, Brazil	-43.4284	-22.9671	155m	AMNH
Ubatuba, São Paulo, Brazil	-45.0867	-23.4444	14m	Surveying
Praia Grande, São Paulo, Brazil	-46.5077	-24.0027	18m	USNM; SI

Table S4: Measures of genetic differentiation: population divergence (%*Dxy*, average number of nucleotide differences per site) between haplogroups calculated for each locus.

	**mtDNA**					**nDNA**	
	**16S**	**12S**	**COI**	**COII**	**CytB**	**CAD**	**EF1α**
***S. cyanea*: NGL *vs* SGL/non-Bahia**	0.42	0.00	2.90	0.14	0.56	0.05	0.00
***S.* aff. *septentrionalis*: NAF *vs* CSAF**	1.42	1.13	1.77	1.93	2.42	0.39	0.19

Table S5: Amova results and *F*st values calculated for each locus based on comparisons among haplogroups, among localities within haplogroups, and within localities.

***S. cyanea*: NGL *vs* SGL/non-Bahia**	**mtDNA**					**nDNA**	
**Percentage of variation**	**16S**	**12S**	**COI**	**COII**	**CytB**	**CAD**	**EF1α**
**Among haplogroups**	57.04	-	59.99	-2.48	43.26	-11.57	-
**Among localities within haplogroups**	38.21	-	23.56	82.05	32.40	32.95	-
**Within localities**	4.75	-	16.45	20.43	24.33	78.62	-
**%*F*st (p-value)**	95.25 (0.000)	-	83.54 (0.021)	79.56 (0.001)	75.66 (0.000)	21.37 (0.438)	-
***S.* aff. *septentrionalis*: NAF *vs* CSAF**	**mtDNA**					**nDNA**	
**Percentage of variation**	**16S**	**12S**	**COI**	**COII**	**CytB**	**CAD**	**EF1α**
**Among haplogroups**	70.92	74.01	67.11	72.18	74.76	28.51	95.44
**Among localities within haplogroups**	25.37	25.99	16.80	25.53	15.67	20.80	-3.74
**Within localities**	3.71	0.00	16.10	2.30	9.56	50.68	8.29
**%*F*st (p-value)**	96.29 (0.000)	100 (0.053)	83.90 (0.005)	97.70 (0.000)	90.438 (0.002)	49.31 (0.036)	91.70 (0.082)

Figure S1: Karyotypes from two armadillo wasps stained with Giemsa. *Synoeca cyanea*: (a) Senhor do Bonfim, BA, (b) Santa Teresa, ES, (c) Carlos Barbosa, RS; and *S*. aff. *septentrionalis*: (d) Moreno, PE, (e) Santa Teresinha, BA, (f) Ilhéus, BA, (g) Vila Regência, ES.
An asterisk indicates specimens with the presence of only one chromosome of a homologous pair.
Two asterisks indicate heteromorphic chromosome pair.
Bar = 10 µm.

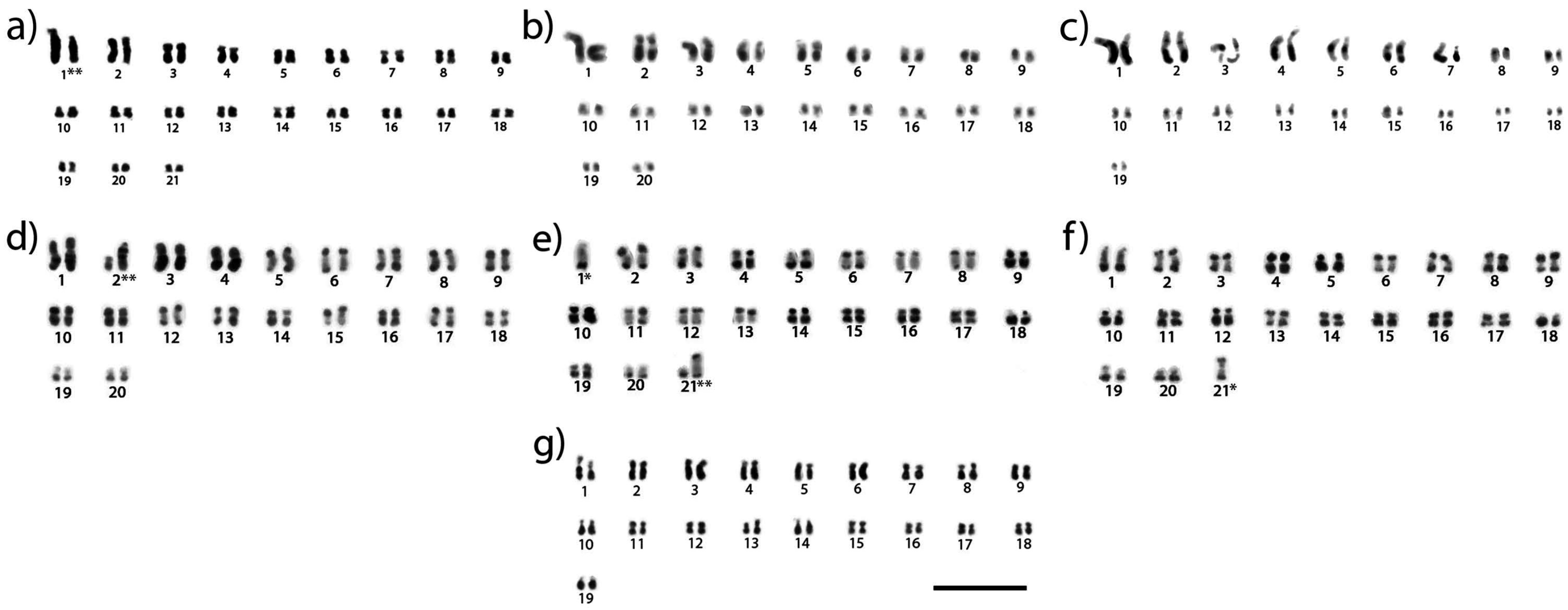

Figure S2: Sequential staining CMA_3/DAPI. *Synoeca cyanea*: (a) Senhor do Bonfim, BA, (b) Santa Teresa, ES, (c) Carlos Barbosa, RS; and *S.* aff. *septentrionalis*: (d) Moreno, PE, (e) Santa Teresinha, BA, (f) Ilhéus, BA, (g) Vila Regência, ES.
Bar = 10 µm.

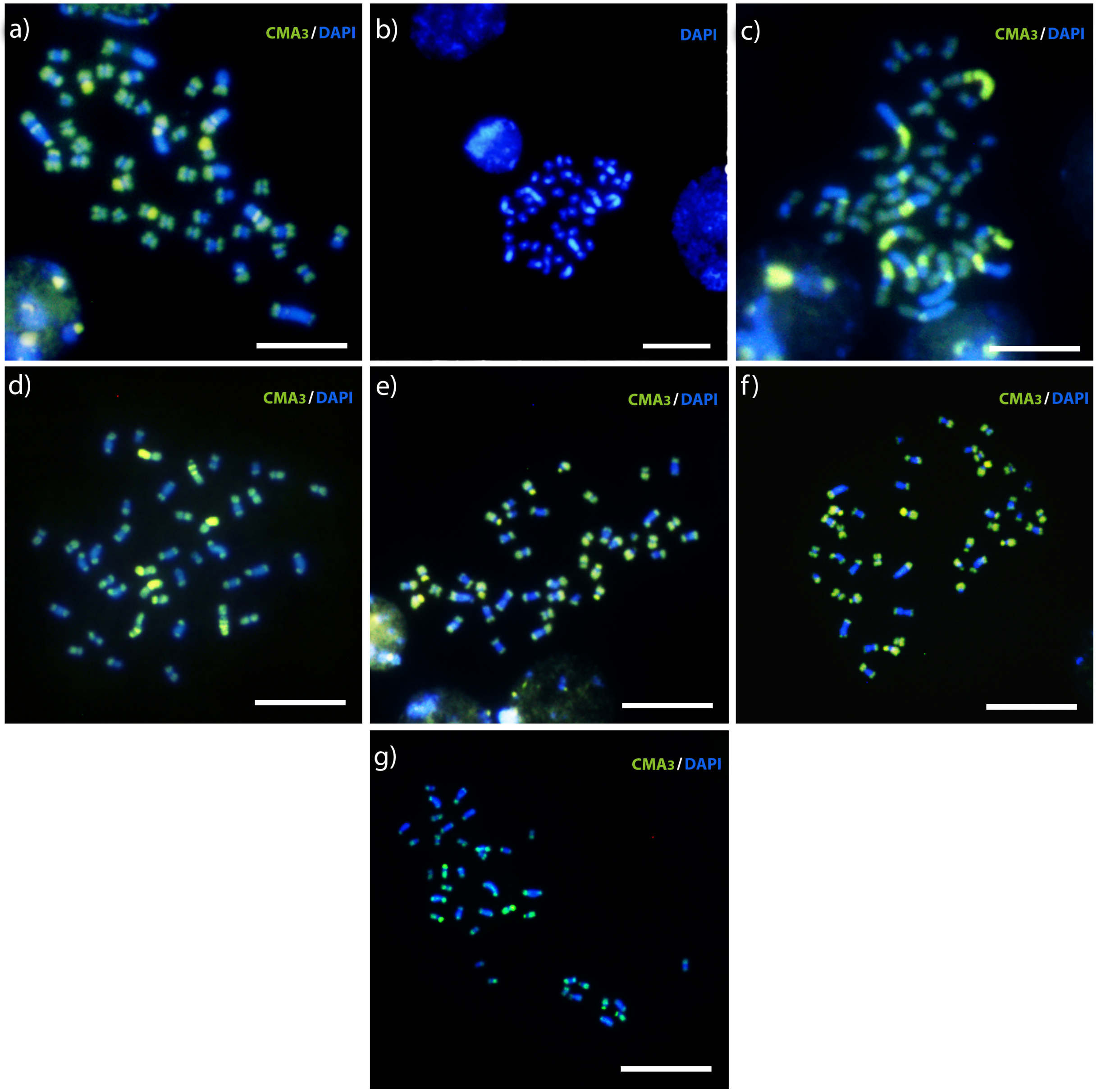

Supplement: Supplementary file 1 — Supplemental materials [file 41598_2017_7776_MOESM1_ESM.pdf]
